# The Impact of Accounting for Future Wood Production in Global Vertebrate Biodiversity Assessments

**DOI:** 10.1007/s00267-020-01322-4

**Published:** 2020-07-05

**Authors:** Katharina Schulze, Žiga Malek, Peter H. Verburg

**Affiliations:** 1 Department of Environmental Geography, Institute for Environmental Studies (IVM), Vrije Universiteit Amsterdam, De Boelelaan 1111, 1081 HV Amsterdam, The Netherlands; 2 Swiss Federal Institute for Forest, Snow and Landscape Research (WSL), Birmensdorf, Switzerland

**Keywords:** Global change, Forest management, Forest plantation, Logging, Land use mapping, Life on Land

## Abstract

Forests are among the most species rich habitats and the way they are managed influences their capacity to protect biodiversity. To fulfill increasing wood demands in the future, planted and non-planted wood production will need to expand. While biodiversity assessments usually focus on the impacts of deforestation, the effects of wood harvest are mostly not considered, especially not in a spatially explicit manner. We present here a global approach to refine the representation of forest management through allocating future wood production to planted and non-planted forests. Wood production, following wood consumption projections of three Shared Socioeconomic Pathways, was allocated using likelihood maps for planted and production forests. On a global scale, plantations for wood production were projected to increase by 45–65% and harvested area in non-planted forests by 1–17%. The biodiversity impacts of changes in wood production patterns were estimated by applying two commonly used indicators: (1) changes in species richness and (2) changes in habitat-suitable ranges of single species. The impact was analyzed using forest cover changes as reference. Our results show that, although forest cover changes have the largest impact on biodiversity, changes in wood production also have a significant effect. The magnitude of impacts caused by changes of wood production substantially differs by region and taxa. Given the importance of forest production changes in net negative emission pathways, more focus should be put on assessing the effects of future changes in wood production patterns as part of overall land use change impacts.

## Introduction

Forests provide key ecosystem services and are crucial for livelihoods of 1.6 billion people, providing them with food and resource security (UN 2018). Sustainable management of forests is necessary to reach a better and more sustainable future for humankind and “life on land” in general, as defined by the Sustainable Development Goal 15 (UN 2018). Although deforestation rates have slowed down in recent years, the forest extent is still decreasing, especially in the species rich tropics (Grooten and Almond 2018; WWF 2015). Global biodiversity is declining at an alarming rate and land cover and use changes are among the most significant contributors to this decline (Butchart et al. 2010; IPBES 2019; Titeux et al. 2016). Forests are indispensable for biodiversity conservation (Brockerhoff et al. 2017), providing habitat for more than 80% of terrestrial species of animals and plants (Aerts and Honnay 2011; Shvidenko et al. 2005). With ongoing deforestation, suitable habitat for forest species is decreasing (Brooks et al. 2002). Other disturbances, such as wood harvest, can substantially change the quality of the remaining forest habitat (Barlow et al. 2016; Chaudhary et al. 2016; Nishijima et al. 2016). The impact of wood harvest can be positive or negative for different species (Chaudhary et al. 2016). However, about the one-third of all forest specialist species and more than half of endangered forest specialist species are seriously threatened by wood harvest due to logging and the establishment of monoculture wood plantations (IUCN 2018b). At the same time, not all forests can support biodiversity to the same extent. Naturally regrown and planted forests usually have lower biodiversity values and cannot serve as replacement for primary forests (Chazdon 2008; Gibson et al. 2011; Laurance 2007; Whitworth et al. 2018). Particularly the species composition might never or only slowly adjust (Gibson et al. 2011; Meyfroidt and Lambin 2011). Planted forests might be only partly able to compensate for the loss of natural forests, as they cannot provide habitats for forest species to the same degree, which is particularly valid for monoculture plantations (Brockerhoff et al. 2008; Brockerhoff et al. 2013; Chazdon 2008). On the other hand, by providing high timber yields on small areas, these plantations are able to reduce harvest pressure on the remaining forests, thereby creating space for conservation areas (Payn et al. 2015). Yet, in order to meet wood demands, it is likely that the share of plantations and their impact on biodiversity will increase in the future (Payn et al. 2015; WWF and IIASA 2012).

The need to halt the biodiversity decline is well recognized in the international community. Biodiversity commitments, such as the Aichi targets (CBD 2010), aim to directly protect biodiversity and/or reduce pressures. Other global commitments, such as the Bonn challenge (IUCN 2018a) or Zero deforestation commitments, (Pasiecznik and Savenije 2017) target forests more directly. They are, however, only useful when considering the types of forests that are lost and the potential of newly afforested areas (Brown and Zarin 2013). Knowing how our use and management of forests might influence biodiversity levels in the future is, therefore, of highest importance.

To study potential future changes in forest cover, land use change models have proven to be helpful tools (Brown et al. 2013; Verburg et al. 2016). Existing modeling studies provide different insights in the role of wood harvests on biodiversity. In global biodiversity hotspots, wood harvest was identified to increase the impact of land use change on biodiversity under different emission scenarios, and could lead to more loss of natural vegetation than agriculture (Jantz et al. 2015). In contrast, a study on fulfilling the Aichi targets globally revealed that the forestry sector only contributes slightly to decreases in species abundance, but the impacts can increase rapidly until 2050 (Kok et al. 2018). Chaudhary and Mooers (2018) calculated global biodiversity loss caused by land use change under different climate and socioeconomic scenarios. They found that the largest loss of natural areas is due to conversion of primary vegetation to secondary vegetation, including forests. Besides these few examples, the effects of future forest use and management remain largely unknown, especially in a spatial explicit context. Assessments of global biodiversity based on future land use scenarios usually only consider the impacts of deforestation. Wood harvest or conversion to planted forests are mostly ignored (Barlow et al. 2016; Chazal and Rounsevell 2009) while, at the same time, most studies treat forests as homogenous habitats. Future impacts on biodiversity could, therefore, be misrepresented.

The aim of this study is to determine the impacts on global biodiversity of both deforestation and wood production. We, thereby, do not aim at a full simulation of land cover and management dynamics. Rather, existing projections of forest extent and global wood demand are used as a starting point. Based on these, we simulate spatial patterns of wood production change and analyze how accounting for these affects biodiversity indicators. We use projections of the latest set of socioeconomic scenarios—the Shared Socioeconomic Pathways (SSPs), consistent with other climate and global change studies (Riahi et al. 2017). Biodiversity impacts are analyzed on aggregated and species level, using biodiversity indicators that are commonly used in global biodiversity assessments (Jetz et al. 2007; Kehoe et al. 2017; Newbold et al. 2016; Powers and Jetz 2019).

## Methods

### Scenarios for Future Land Use and Wood Consumption

The SSPs are the most commonly used set of socioeconomic storylines, created as a community effort by the climate change research community (Riahi et al. 2017). The five different SSP storylines describe potential challenges for climate change mitigation and adaptation and range from low, over middle to high challenges (O’Neill et al. 2017; Riahi et al. 2017). Three scenarios were taken into consideration: SSP1, SSP2, and SSP3, presenting a range of different consumption patterns and socioeconomic developments. SSP4 and SSP5 were not included, as they are ‘asymmetric’ scenarios and their results are expected to be in the range of the other scenarios. Furthermore, consistent global projections on wood consumption and future land use for these scenarios are not available. SSP1, as the ‘Sustainability’ storyline, describes future development with more resource-sparing lifestyles, low increases in population and all-in-all low pressures on land (van Vuuren et al. 2017). SSP2 represents the “Middle of the Road” pathway, characterized by a modest population growth and a slight decrease of resource use intensity. Regional rivalry in form of regional conflicts and a remerged nationalism is represented in SSP3 and comes with high challenges to climate change mitigation and adaption (Fujimori et al. 2017). Compared to the other two scenarios, higher resource use intensities, population growth in developing countries and higher rates of deforestation are projected (Fujimori et al. 2017; O’Neill et al. 2017). In this study, we focused only on the baseline climate scenarios of each SSP. The baseline scenarios project future development without newly introduced climate policies (Riahi et al. 2017). Climate change in 2050 is thus represented by Representative Concentration Pathway 4.5 for SSP1 and SSP2 and 6 for SSP3. The term ‘baseline scenario’ should not be confused with the term ‘marker scenario’, which refers to implementation of also a specific Integrated Assessment Model (Riahi et al. 2017).

### Area of Wood Production

In the following sections, we describe the calculation of future changes of planted and non-planted wood production forests and their spatial allocation. We adopted the definitions by FAO’s Global Forest Resource Assessment, which the spatial dataset from Schulze et al. (2019) is based on. Planted wood production forests are defined as forest areas with more than 50% of trees deliberately planted or seeded and designated to be used primarily for wood production (MacDicken 2012). Non-planted wood production forests are defined as forests with more than 50% of trees naturally regrown and designated to be primarily used for wood production (MacDicken 2012).

The calculations of required areas for wood harvest were based on roundwood consumption, using existing data from an Integrated Assessment Model for the SSP scenarios (Doelman et al. 2018; van Vuuren et al. 2017). For the current extent of production and plantation forest, we used existing maps of forest classes and uses (Schulze et al. 2019). New areas, necessary to fulfill future wood consumption, were allocated using likelihood maps for forest management types based on location factors following Schulze et al. (2019). All calculations were done using R (R Core Team 2018) and ArcMap (ESRI 2016). The next sections describe in more detail each step of the method for projecting future wood production areas.

### Wood Consumption

Wood consumption for the year 2050 followed the projections for SSP1, SSP2, and SSP3 available from the Integrated Model to Assess the Global Environment (IMAGE) (Doelman et al. 2018; van Vuuren et al. 2017). These projections provide total roundwood consumption and the shares of industrial roundwood and wood fuel for 26 world regions (ESM section 1.3, Stehfest et al. 2014). In the model, which the dataset stems from, wood fuel projections are based on an energy model and take access policy, poverty and the size of the rural population into account (Doelman et al. 2018). The consumption of industrial roundwood is in the model determined by multiplying population projections with estimates of capita consumption, which depend on the scenario as specified in the IMAGE model implementation (Doelman et al. 2018). In SSP1, capita consumption reduces by 10%, while it increases by 5% and 10% in SSP2 and SSP3, respectively (Doelman et al. 2018). For consistency, we also used the data on current (2005) wood consumption from this dataset.

It is not clear, to what extent wood harvested during deforestation accounts toward reported roundwood consumption (Hurtt et al. 2011; Smeets and Faaij 2007). Some existing land change studies consider this wood as part of reported roundwood consumption (Kok et al. 2018; McGrath et al. 2015) while others do not (Hurtt et al. 2011; Stocker et al. 2014). We included wood harvested during deforestation as a means to fulfill wood demand, considering the significant relationship between deforestation and timber harvest (Damette and Delacote 2011) and following a study using the same roundwood consumption scenarios in a global integrated assessment (van der Esch et al. 2017).

To estimate the amount of forest biomass available through deforestation, we spatially overlaid global data on forest loss in 2005 (Hansen et al. 2013) with wood biomass density (Avitabile et al. 2014, 2016), and converted the results from density to volume (Santoro et al. 2015). Only loss areas outside the forest extent of this study (described below) were included to avoid double counting of clear-cut forestry areas. The share of annual burnt area (Giglio et al. 2013) was subtracted from the amount of timber available from deforestation, since fires are often used for land conversion and uncontrolled wildfires are responsible for a major share of forest loss in many world regions (Curtis et al. 2018). Partially, wood from burnt forest is still harvested, either as pre-harvest before the burning or afterward through post-fire salvage logging. However, as the amount of wood from these harvests is unknown, we did not consider them in our calculations, but assumed all wood from burnt forests as unusable. Depending on the region, different shares of roundwood from deforestation were taken into account (ESM section 1.5). The amount available through deforestation was then subtracted from the total consumption. The remaining roundwood consumption was presumed to equal the amount produced in production forests. Losses, for example in the production and supply chain, are assumed to be accounted for in the underlying data and therefore not considered. To account for the different shares of wood harvested from planted and non-planted production forests, we used values from d’Annunzio et al. (2015), which are based on estimates from national experts.

We used existing global land use scenarios to determine future forest extents (Wolff et al. 2018). These scenarios have been simulated by the CLUMondo model, based on the demand of different goods and services, following the same SSP storylines. The model allocates different land systems, necessary to fulfill future demands, by taking into account socioeconomic and biophysical characteristics (van Asselen and Verburg 2013). Future wood available through deforestation was calculated from the forest loss resulting from land conversion projected in the Wolff et al. (2018) scenarios, using the same parameters as for the current wood consumption. The same share of deforestation due to fire for the year 2005 was applied for the future.

### Current Wood Production Areas

To determine the area of current planted and non-planted wood production forests, we harmonized the maps of current forest classes and forest uses (Schulze et al. 2019) with the global map of land use systems used in the CLUMondo model (van Asselen and Verburg 2012, see ESM section 1.1). All final analysis was conducted on a 1 × 1 km^2^ resolution.

The current extent of planted forest was split into productive and non-productive plantations by using regionally specific average shares (Del Lungo et al. 2006, ESM section 1.8). In contrast to productive plantations, non-productive plantations are established to provide non-commercial services, such as water and soil conservation (Palmberg-Lerche et al. 2001). Productive plantations were spatially allocated within the planted forest extent using the production forest likelihood maps from Schulze et al. (2019) (ESM section 1.7 and Fig. S_4). The area of non-planted production forests was obtained by summing all forest areas primarily designated for production, excluding the area of productive plantations. In 17 countries, no forests are designated to be primarily used for production (ESM section 1.6). In such countries, 30% of forest areas used for multiple purposes was assigned as production area, following d’Annunzio et al. (2015). The maps by Schulze et al. (2019) were based on data for the year 2000, but wood consumption was given for the year 2005. Therefore, the extent of planted and non-planted production was adjusted by the change between 2000 and 2005 from the Global Forest Resource Assessment (FAO 2016).

### Wood Yields and Future Area

Wood production per area (i.e. yield) was derived by dividing wood from planted or non-planted forests with the spatial extents of the production area. For the future, we increased the share of wood from productive plantations and wood yields of these areas, following projections on wood production from plantations (d’Annunzio et al. 2015). Potential productivity changes in non-planted production forests were not accounted for, as data on trends in production are lacking (Table S_4). Finally, the area of planted and non-planted forests needed for future wood consumption was calculated by dividing wood consumption with future yields of production forests.

### Allocation of New Areas

For the allocation of additional planted and non-planted areas, we used maps depicting the likelihood of finding these forest types. These likelihood maps were prepared by Schulze et al. (2019) separately for four different biomes, with the help of regression models. These models estimated forest management based on location characteristics and observational points representing local forest management. Location characteristics included accessibility indicators, soil, and environmental properties, terrain variables and forest conditions (see Table S_1). Following the methodology from Schulze et al. (2019), we updated planted forest likelihood maps for regions with dense national or subnational data coverage, namely Brazil, Canada, Oceania, Russia, USA, and Western Europe (documented in ESM section 1.7). Wood production areas, additionally needed to fulfill future wood demands, were then allocated in forest areas with the highest likelihood values until the demand was met. Areas for planted wood production were thereby allocated first, followed by areas for non-planted wood production.

### Effects on Biodiversity

**Fig. 1:**
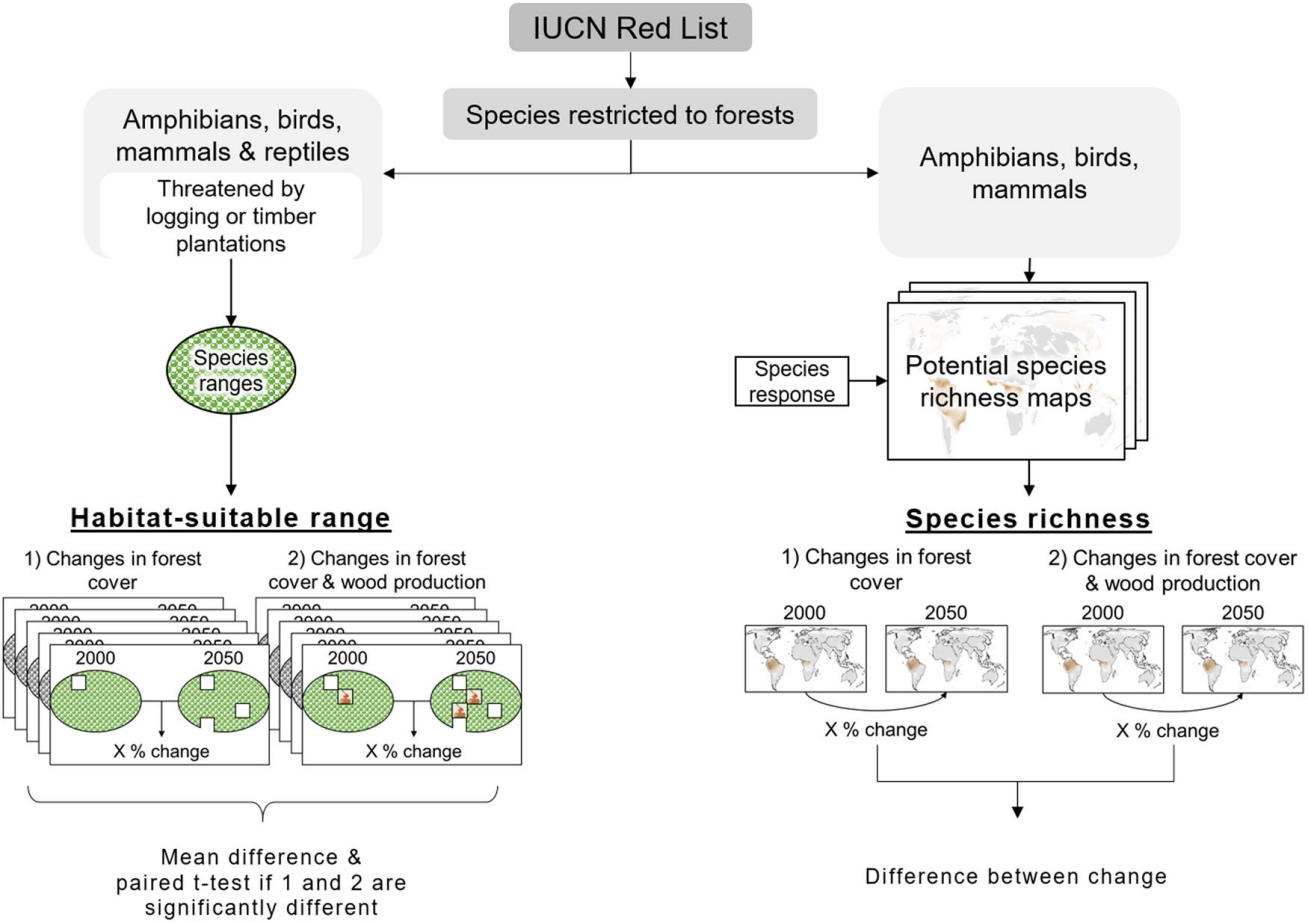
Methodology for estimating the impact of wood production on biodiversity indicators. The differences due to forest cover changes were compared to the differences due to both, forest cover and wood production changes. Only forest specialist species were considered for both indicators. Forest and wood production areas were overlaid with potential species richness maps based on habitat range polygons. For habitat-suitable ranges, areas of wood production and timber plantations were assigned as unsuitable for threatened species. For estimating species richness, species response values for wood production types from Chaudhary et al. (2016) were assigned to forests with wood production

The impacts of accounting for wood production on biodiversity assessments were studied by assessing two biodiversity indicators: (1) habitat-suitable ranges (HSR) for single species and (2) global and regional forest species richness, expressed in mean ratio. Both indicators are frequently used in global biodiversity assessments, since they are easy to understand, measure, and calculate (Lamb et al. 2009). Recent studies adopting these indicators include Jetz et al. (2007), Newbold et al. (2016), Kehoe et al. (2017), and Powers and Jetz (2019). Using an indicator for single species and an indicator, which aggregates across species, enables us to assess the impacts on biodiversity on different levels. For both indicators, the relative changes between 2000 and 2050 for the three scenarios were assessed (see Fig. 1), including existing, as well as, new forested areas. The indicators were compared for the situation where only changes in forest cover were considered to the situation in which also wood production was accounted for. Only forest specialist species, i.e. those species that do not occur outside of forests, were included and queried from the Red List database (IUCN 2018b). Species extinct in the wild were excluded. Both biodiversity indicators were analyzed globally and for seven major world regions separately (ESM section 1.3).

The impacts on HSR were calculated for amphibian, bird, mammal, and reptile forest specialist species listed in the IUCN Red List database as threatened by logging or timber plantations. A species can be threatened by logging through the reduction of habitat quality, for example by changing tree density, horizontal forest structure or species composition. The same accounts for timber plantations, where the changes are additionally more abrupt, leaving less time for species to adapt. It was assumed that wood production or planted wood production areas, respectively, do not represent suitable habitat for these species ranges. To assess the changes in HSR, we used spatial data on species habitat range (BirdLife International and Handbook of the Birds of the World 2017; IUCN 2018b). Since species range data are overestimating the actual occurrence of species, it has been suggested to apply it only on coarse resolution of 1°, i.e. ~110 × 110 km^2^ (Hurlbert and Jetz 2007). The uncertainties that occur on higher resolution assessments can, however, be addressed and reduced to a minimum by excluding unsuitable habitat, as demonstrated by Jetz et al. (2007) and Powers and Jetz (2019). We followed a similar approach by focusing solely on forest areas and only including forest specialists, i.e. species that do not occur outside of forests. Each species’ habitat range was overlaid with the total forest extent, as well as, with the spatial extent of suitable habitat. Suitable habitat was thereby estimated by using the total forest extent within the species range and excluding areas of wood production or planted wood production, respectively. The relative HSR changes for these two measurements were then compared. To test significance of changes in HSR, paired *t* tests were applied. The indicator was aggregated, by calculating the average of each species’ relative change. As a result of our assumptions, the extent of wood production areas and the HSR had here always a negative relationship, meaning that an increase in the extent of wood production will decrease the HSR of the species under consideration. To understand the trajectories of HSR without changes in wood production, we furthermore calculated the HSR for all forest specialist species for the year 2000 and 2050, solely based on forest cover.

Species richness was calculated for amphibian, bird, and mammal forest specialist species. Reptiles were here excluded, as data were not available. The selection of taxa was based on data availability of species range (BirdLife International and Handbook of the Birds of the World 2017; IUCN 2018b) and species response to forest management types (Chaudhary et al. 2016). Changes in species richness were determined by overlaying potential species richness maps with our spatial results on forest cover and wood production changes. Potential species richness maps were created for mammals, amphibians and birds, based on habitat ranges for single species (BirdLife International andand Handbook of the Birds of the World 2017; IUCN 2018b). To evaluate the impact of changing planted and non-planted wood production, we used values from a review on the impacts of different forest management types on species richness (Chaudhary et al. 2016). When available, species response values per management, taxa and continent were distinguished. When no separate values for a continent was given, a global average was used (Table S_3). To harmonize categories between Chaudhary et al. (2016) and our study, mean response ratios for planted and non-planted wood production forests were calculated. For planted production forests, we averaged response ratios for plantation fuel and plantation timber (Table S_3). For non-planted production forests, species responses were weighed with shares of harvest type, e.g. clear-cut or selective logging (Arets et al. 2011). According to the findings by Chaudhary et al. (2016), as opposed to our HSR calculations, wood harvest can have positive or negative impacts on species richness, depending on the taxa, region, and wood production type. For example, amphibians are positively affected by non-planted wood production in South America and Africa, which can be related to improved reproduction conditions (Semlitsch et al. 2009). For mammals, species richness increases in non-planted wood production of four regions, which is probably due to better grazing conditions and food availability, e.g. in form of seeds or smaller mammals (Wearn et al. 2017). For all taxa-region combinations, planted wood production decreases species richness. The species richness change caused by forest cover changes was calculated by assuming original species richness within forests and no species richness outside of forests. Due to the larger set of included species (threatened and non-threatened) and the consideration of positive and negative impacts on species, the impact of wood production will show a less negative impact on species richness, compared to the HSR.

## Results

### Future Extent and Location of Planted and Non-planted Wood Production Areas

**Fig. 2:**
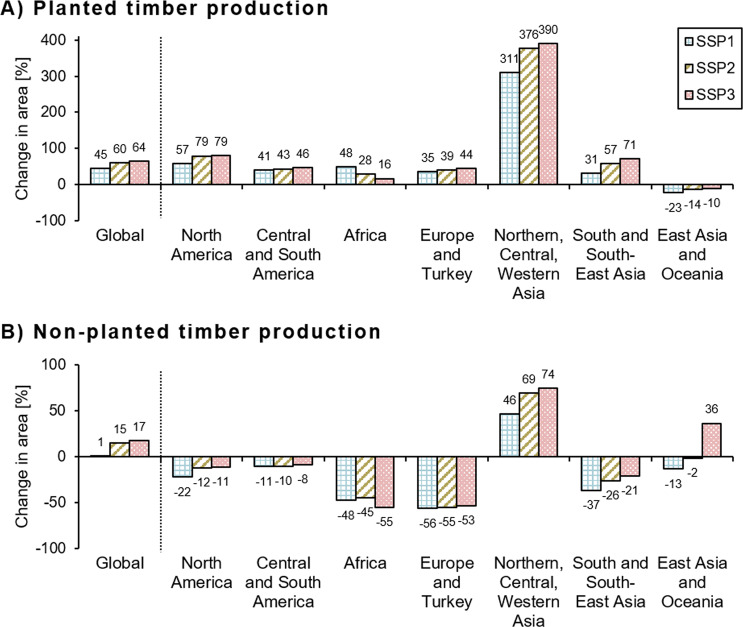
Relative changes of planted (**a**) and non-planted (**b**) timber production areas globally and in seven major world regions, following three SSP scenarios

To fulfill future global wood consumptions, the extent of forest used for wood production was projected to expand by 7% for the SSP1 scenario, 21% for SSP2, and 24% for SSP3 as compared to the extent of wood production in 2000. Planted wood production forest area was determined to increase by about 45–65% of the initial extent in 2000 (~500–700,000 km^2^). The SSP3 scenario showed the largest increment, followed by SSP2 and SSP1 (Fig. 2). The share of wood produced in planted forest increased from 4% in 2000 to 5–7% in 2050. The area of wood production in non-planted forests shrank in most major regions. However, mostly due to an increase in Northern, Central and Western Asia region and especially Russia, the global area expanded still by 1–17% (depending on the scenario) of the area of wood production in 2000. Only in the region of Northern Central and Western Asia, planted and non-planted wood production areas were both projected to expand in all scenarios (Fig. 2, Fig. S_5 and Table S_4).

**Fig. 3:**
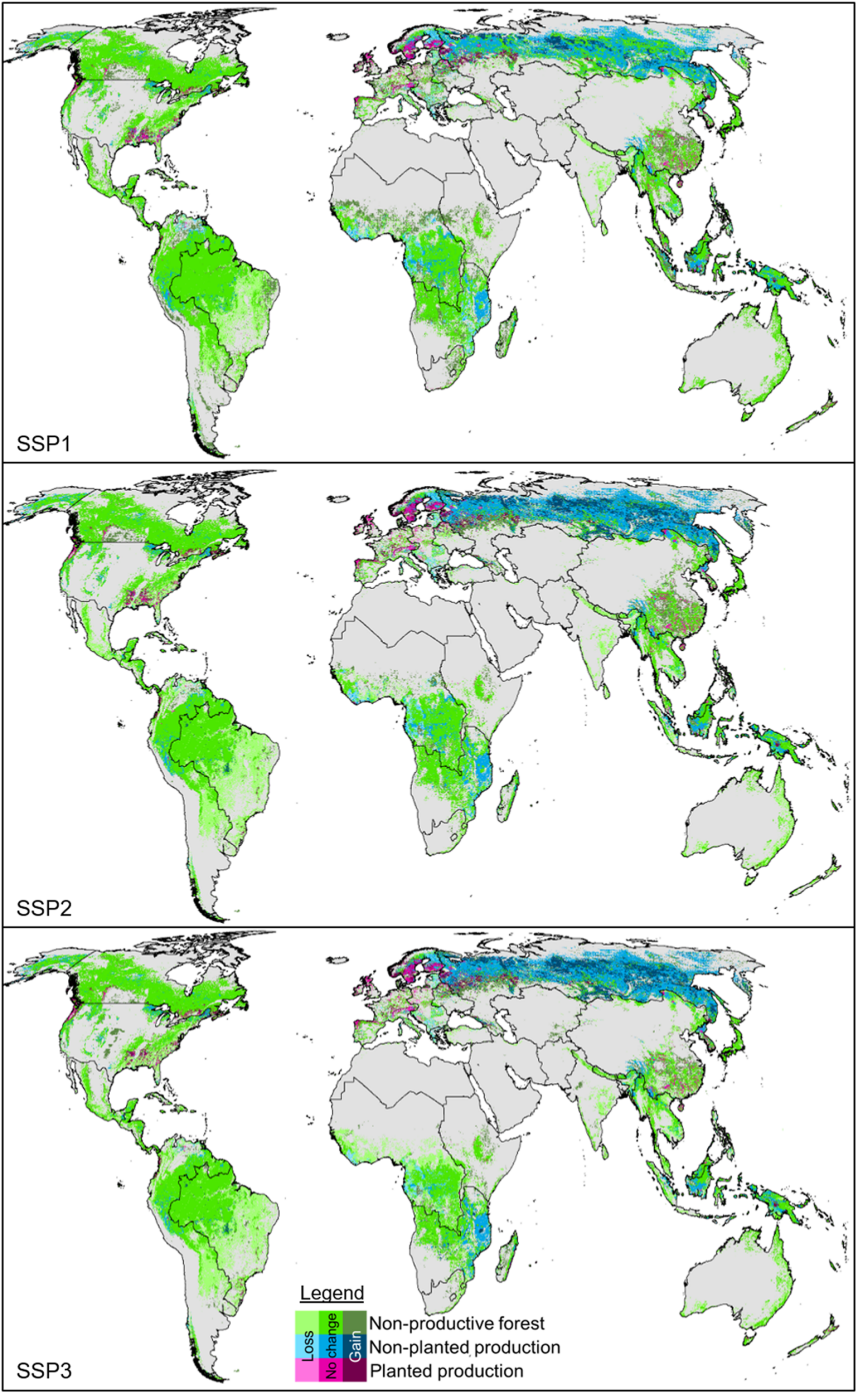
Projected distribution of forests and timber production areas in 2050 compared to 2000 following wood demand projections from three SSP scenarios. The extents in the year 2000 are listed in Table S_1

Planted wood production increased substantially in North America, South and South-East Asia and Europe and Turkey. At the same time, in South and South-East Asia, as well as, Europe and Turkey the largest relative declines of non-planted wood production areas were found. In absolute numbers, non-planted wood production shrank the most in Africa. East Asia and Oceania was the only region where the extent of planted wood production was projected to decrease, mostly in China. At the same time, this region showed an expansion of non-planted wood production in the SSP3 scenario. New areas of wood production were allocated mostly close to existing areas. New planted forests were mostly established on former natural regrown forests (~80% in all scenarios) and around 20% in former primary forests (Fig. 3).

### Effects on Biodiversity

We evaluated the impact of wood production on species richness and HSR. Therefore, the change of indicator values between 2000 and 2050 were compared when wood production was considered and when only forest cover changes were taken into account. The difference between the changes was then regarded as the impact of wood production on the biodiversity indicator. The impacts on species richness are in the following expressed in ratios of overall change, while the impacts on HSR are expressed in mean over species’ range ratios.

**Fig. 4:**
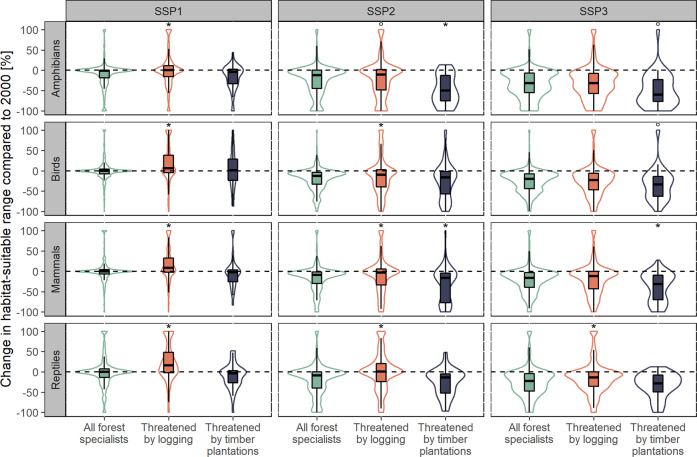
Changes in habitat-suitable ranges. Violin-boxplots represent the relative changes of suitable habitat for reptile, mammal, bird, and amphibian species threatened through timber plantations (blue—right) and logging (orange—middle), when considering forest cover changes and wood production. The green plot (left) shows the changes of all forest specialist species (threatened and non-threatened), when only forest cover changes were considered. *T* tests were applied to estimate if the relative changes for species threatened by logging or timber plantations are significantly different from the relative changes of all forest specialists, considering only forest cover changes. Significance levels are thereby given above the violin plot (*p < 0.01, °*p* < 0.05, no symbol: not significant). Note: Values were truncated at 100% for better visualization, thus resulting in higher kernel densities for value 100%

On a global scale, forest cover changes did on average have no effect on HSR values of all forest specialist species (combining threatened and not-threatened by wood production) between 2000 and 2050 in the SSP1 scenario, while the indicator decreased for these species in SSP2 and SSP3 (boxplots in Fig. 4). When accounting for wood production, HSRs of species threatened by logging increased significantly more from 2000 to 2050 in SSP1 and decreased significantly less in SSP2, compared to the changes of all forest specialists resulting from forest cover changes. Reptiles had, thereby, the largest magnitude of change (Fig. 4). For the SSP3 scenario, solely HSR changes of reptiles threatened by logging were significantly different from the changes of all forest specialists. For amphibian, bird, and mammal species threatened by timber plantations, compared to forests specialists, HSRs showed a significantly larger decrease in the SSP2 and SSP3 scenarios (Fig. 4). For the other scenario-taxa combinations, the difference was not significant on a global scale. Violin plots (Fig. 4) represent the kernel density and provide an indication for the trend and distribution of impacts across species. SSP1 points toward more localized effects, affecting single species, depending on their specific range location. This results in a more even distribution of HSR losses and gains, which is demonstrated by rather narrow violin plots and values clustered around 0. On the contrary, more widespread loss of HSR was projected in SSP3, especially for species threatened by timber plantations, as demonstrated by much wider violin plots with more values clustered below 0 (Fig. 4).

Paired *t* tests indicated if the HSR changes between 2000 and 2050 were significantly different when the area changes of logging or timber plantations were included, as opposed to when solely considering forest cover changes. Accounting for changes in planted and non-planted wood production patterns had globally for all scenarios a significant positive impact on species threatened by logging, caused by the reduction of wood production areas in most major regions due to more production in planted areas with a higher yield and hence a smaller extent. An exception are reptiles (in all scenarios), as well as, birds, and amphibians in SSP3. For these taxa-scenario combinations, there were no significant differences between solely considering forest cover changes and considering also wood production (Table 1). Changes in planted wood production had globally a significant negative impact on mammal species threatened by timber plantations in the SSP1 scenario, amphibians, birds, and mammals in the SSP2 scenario and birds and mammals in the SSP3 scenario. Comparing the changes of median HSRs between regions showed a similar picture. In most regions and scenarios, when including planted and non-planted wood production, the average HSR decreased significantly less or expanded significantly more, compared to only considering forest cover changes (Table 1), resulting in a positive impact on species threatened by logging. Mammals, threatened by logging, in North, Central, and Western Asia was the only taxa-region combination with a negative impact on median HSRs in all scenarios, with the largest impact in the SSP3 scenario (77% median difference compared to forest cover changes only). For species threatened by timber plantations, the impacts of changes in planted wood production always caused the median HSR to decrease more than when only forest cover changes were considered.

**Table 1:** Changes in habitat-suitable ranges (HSR) for species threatened by logging or by timber plantations in scenarios considering wood production and forest cover changes in relation to scenarios that solely consider forest cover changes

Region	Species threatened by logging							Species threatened by timber plantations						
		SSP1		SSP2		SSP3			SSP1		SSP2		SSP3	
	*n*	Imp.	M∆ % *p*	Imp.	M∆ % *p*	Imp.	M∆ % *p*	*n*	Imp.	M∆ % *p*	Imp.	M∆ % *p*	Imp.	M∆ % *p*
Amphibians														
Global	513	pos.	0.1^•^	pos.	0.0^•^	pos.	0.0^n.s.^	36	neg.	0.0^n.s.^	neg.	0.0^•^	pos.	0.0^n.s.^
North America	108	pos.	0.0*	pos.	0.0^•^	pos.	0.0^n.s.^	2	–	–	–	–	–	–
	248	pos.	0.0^n.s.^	pos.	1.4^•^	pos.	0.0^n.s.^	28	neg.	0.0^n.s.^	neg.	−0.8^•^	neg.	−2.8*
Central and South America														
Africa	92	pos.	7.0*	pos.	0.1*	pos.	0.0^n.s.^	2	–	–	–	–	–	–
South and South-East Asia	63	pos.	4.1*	pos.	−4.6*	pos.	0.0^n.s.^	4	–	–	–	–	–	–
East Asia and Oceania	21	pos.	0.0^n.s.^	pos.	0.6^n.s.^	pos.	−0.9^n.s.^	0	–	–	–	–	–	–
Birds														
Global	657	pos.	4.8*	pos.	0.6*	neg.	0.0^n.s.^	108	neg.	0.0^n.s.^	neg.	−1.2^•^	neg.	−1.1*
North America	34	pos.	1.3*	pos.	0.6*	pos.	0.1^n.s.^	2	–	–	–	–	–	–
	203	pos.	0.0*	pos.	0.0^n.s.^	pos.	−0.2^n.s.^	33	neg.	−0.4*	neg.	−3.0*	neg.	−2.6-
Central and South America														
Africa	68	pos.	11.8*	pos.	3.9*	pos.	0.0^n.s.^	5	–	–	–	–	–	–
South and South-East Asia	309	pos.	16.4*	pos.	3.2*	pos.	0.0^n.s.^	59	neg.	−0.2*	neg.	−3.2*	neg.	−1.3*
East Asia and Oceania	77	pos.	0.6*	pos.	0.0^•^	pos.	0.0^•^	11	pos.	6.4^n.s.^	pos.	6.2^n.s.^	pos.	3.2^n.s.^
Mammals														
Global	606	pos.	9.1*	pos.	2.2*	pos.	0.0*	58	neg.	0.0^•^	neg.	−0.3*	neg.	−0.6*
North America	42	pos.	1.1*	pos.	0.3^•^	pos.	0.3^n.s.^	0	–	–	–	–	–	–
Central and South America	138	pos.	0.0*	pos.	−0.2^•^	pos.	−0.2^n.s.^	13	neg.	−3.7*	neg.	−3.6*	neg.	−4.8*
Africa	196	pos.	15.0*	pos.	3.8*	pos.	1.2*	7	pos.	0.0^n.s.^	pos.	−0.3^n.s.^	pos.	−0.1^n.s^
North, Central, Western Asia	10	neg.	−33.0*	neg.	−69.3*	neg.	−76.7*	2	–	–	–	–	–	–
South and South-East Asia	201	pos.	11.5*	pos.	2.6*	pos.	0.4^n.s.^	31	neg.	0.0^n.s.^	neg.	−0.2^n.s.^	neg.	−0.6^n.s^
	72	pos.	28.9*	pos.	28.3*	pos.	8.1*	9	pos.	1.0^n.s.^	pos.	0.1^n.s.^	pos.	0.0^n.s.^
East Asia and Oceania														
Reptiles														
Global	457	pos.	7.9^n.s^	pos.	2.1^n.s.^	pos.	0.1^n.s.^	46	neg.	0.0^n.s.^	neg.	0.0^n.s.^	neg.	0.0^n.s.^
North America	32	pos.	0.4*	pos.	0.2*	pos.	0.2*	2	–	–	–	–	–	–
Central and South America	124	pos.	0.7^n.s.^	pos.	0.1^n.s.^	pos.	0.0^n.s.^	7	neg.	0.0^n.s.^	neg.	0.0^n.s.^	neg.	0.0^n.s.^
Africa	185	pos.	12.0*	pos.	2.6*	pos.	−0.5^n.s.^	10	neg.	0.0^n.s.^	neg.	0.0^n.s.^	neg.	0.0^n.s.^
South and South-East Asia	111	pos.	21.6*	pos.	10.4*	pos.	0.3^n.s.^	19	pos.	0.0^n.s.^	pos.	−0.6^n.s.^	pos.	−0.4^n.s.^
East Asia and Oceania	34	pos.	41.2*	pos.	53.4*	pos.	17.5*	10	pos.	0.0^n.s.^	pos.	−5.4^n.s.^	pos.	0.0^n.s.^

Paired *t* test were applied to test if scenarios that considered wood production were significantly different from scenarios considering only forest cover changes. A positive impact (pos.) means that in scenarios that included wood production, mean ratios of HSR decreased less (or increased more) compared to scenarios, where only forest cover changes are considered. A negative impact (neg.) means mean ratios of HSR decreased more, or the increase was smaller

Note: Regions with less than five species were excluded. Regions with less than five species threatened by logging and timber plantations are not listed in the table (Europe and Turkey for all taxa and North, Central, Western Asia for all taxa, besides mammals)

*n* number of species, *Imp.* impact on the mean ratio, *MΔ %* median difference in percent, *p* significance level

*p < 0.01; ^•^*p* < 0.05; ^n.s.^*p* > 0.05 (not significant)

**Fig. 5:**
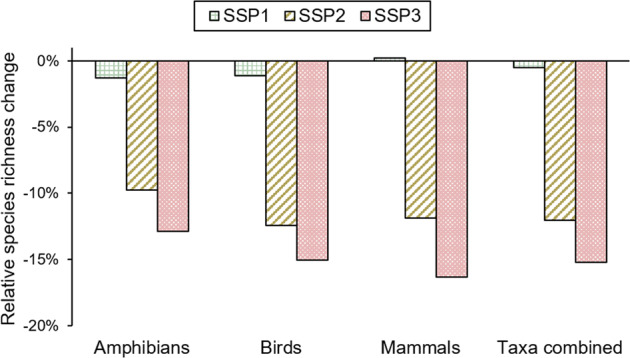
Change in global species richness of amphibians, birds, mammals, and the three taxa combined in 2050, following 3 SSP scenarios, relative to the year 2000

**Fig. 6:**
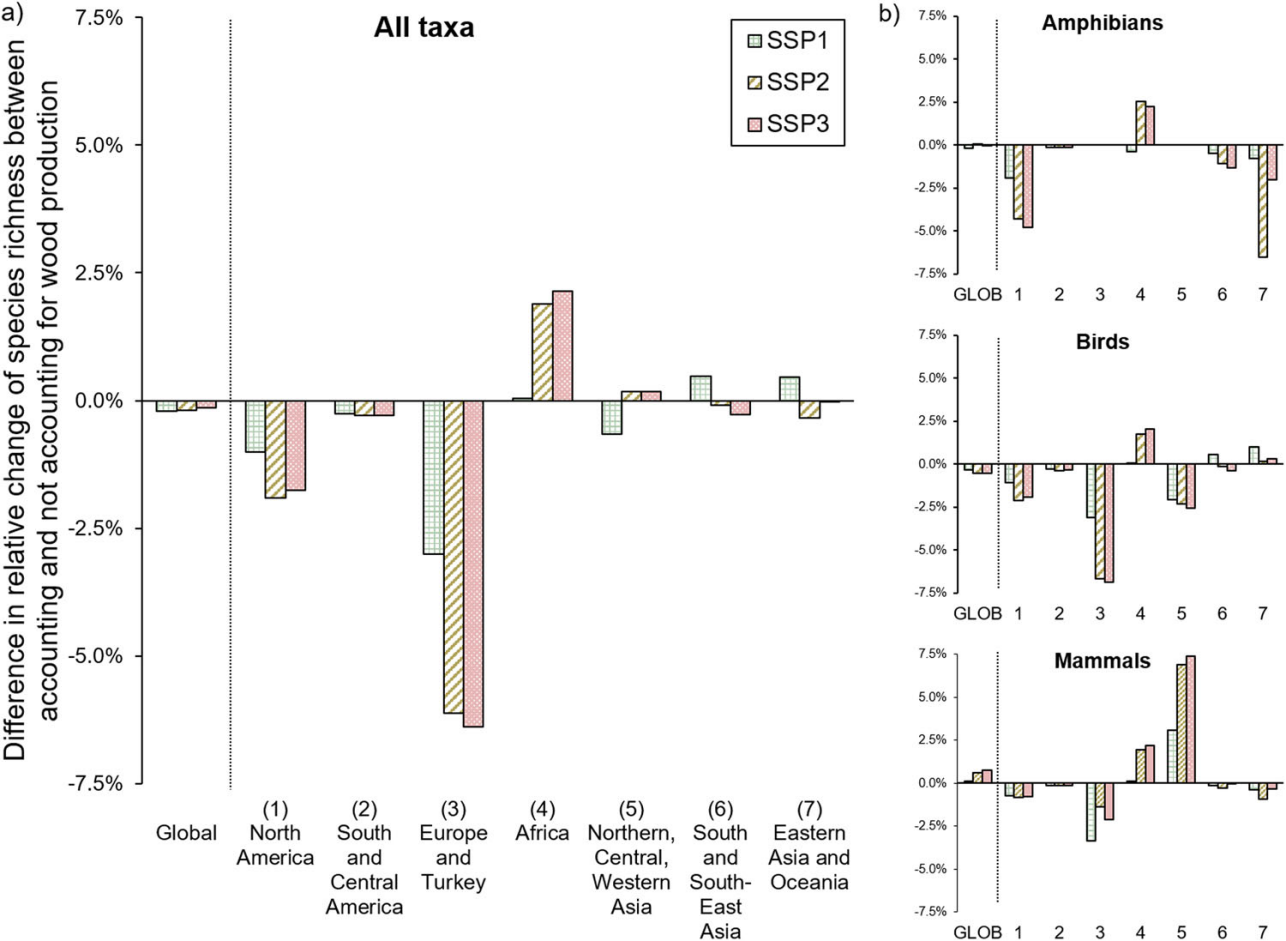
Impact of wood production on potential species richness between 2000 and 2050 when compared to calculations only considering forest cover changes

When combining both forest cover changes and wood production, species richness declined globally for all taxa combined in all scenarios (Fig. 5 and Table S_5). Likewise, wood production alone had a negative impact on global species richness in all scenarios for all taxa combined. The impact was, however, in all scenarios below 1% for the taxa combined (see Fig. 6a). For bird species, the impact was slightly more negative, but still in all scenarios below 1%. On the contrary, wood production had in all scenarios a slightly positive impact on mammal species richness. On a regional scale, impacts by wood production on species richness were more distinct. In Europe and Turkey, negative impacts on the richness of all species combined (~6%), as well as of birds and mammals (~7% and ~3%, resp.) were for all scenarios the largest (compared to the other regions). This is caused by the expansion of planted wood production. In Africa, positive impacts for all species were determined in all scenarios (between 1 and 3%), which was caused by the reduction of non-planted wood production area. In all scenarios for Northern, Central, and Western Asia, the positive impact of wood production on mammal species richness stood in contrast to the negative impacts on bird species richness (see Fig. 6b).

## Discussion

### Location Matters

By combining existing projections of wood consumption with existing land cover simulations, we created global and regional projections of future planted and non-planted wood production extents and their spatial allocation for three SSP scenarios. Generally, the smallest extent of production forest was found for the SSP1 scenario and largest for SSP3. This shows that increase of wood available by converting forests to agricultural areas or other land cover types, cannot compensate for increased global wood consumption in these scenarios. Our projected increase in wood production forest extents are within the range of other global studies. They are about twice as high as the projections from a report on global land change for the same scenarios (van der Esch et al. 2017). This can be explained by the fact that van der Esch et al. (2017) used higher estimates on informal production of wood fuel and the share of wood from planted forests in the future. Compared to the results of another future global land use change study by Kok et al. (2018), our wood production extents are about 50% smaller. In this case, the change is due to the different applied scenarios. Regional differences on projections for wood production patterns are not included in these studies and, therefore, our results cannot be compared in more detail.

The large increase of non-planted wood production forests in Russia dominates our results. However, even in the current situation Russia has by far the largest estimated area of wood production, five times larger than, for example, the USA (FAO 2016). The amount of roundwood currently produced in Russia, on the other hand, is compared to the USA, four times lower. Hence, the calculated timber yield is comparably low, which can be attributed to unfavorable environmental conditions such as more extreme climate, excessive soil humidity or permafrost, as well as overexploitation of forests that used to have higher yields (FAO 2012). According to wood consumption data, roundwood production in Russia will increase by up to 75% until 2030 (amounting totally to still 3 times less than the USA). While this is a relatively high increase compared to the other regions, it is low compared to other scenarios, which project an increase by up to 200% (FAO 2012).

Forest plantations can be used to relieve the logging pressure from natural forests and will be necessary to fulfill future wood demands (Payn et al. 2015; Pirard et al. 2016; WWF and IIASA 2012). However, by replacing primary forests, plantations substantially change habitat conditions and species composition (Bremer and Farley 2010; McFadden and Dirzo 2018). In our study, about 20% of newly planted wood production areas were projected to occur in former primary forests. Interestingly, only negligible amounts of planted and non-planted wood production were projected on areas of new forest gain until 2050. This may be linked to the fact that the likelihood maps (which the allocation was based on) were produced for current conditions and do not account for changes in climate, accessibility, or other variables. On the other hand, it may be an indication that new forest areas are less suitable for planted forests, and thus less productive. Often new forests grow on abandoned marginal farmlands in mountain regions, not necessarily suitable for productive forest plantations. Commitments aiming at no net forest loss or afforestation, therefore, should carefully consider the value of new forested areas compared to those that are lost (Bremer and Farley 2010).

Our results highlight the importance of including wood consumption and production patterns in studies that analyze the interactions and feasibility of global targets. For example, the Sustainable Development Goal 15 urges for sustainable forest management and a stop to forest loss to protect biodiversity (UN 2018). Achieving this goal, however, competes for land with other goals, such as Zero Hunger (Goal 2) in Sub-Saharan Africa (Nilsson et al. 2016). As our study has demonstrated, more forested land will be required globally to fulfill future wood demand. In Africa, planted, hence, intensive wood production was projected to increase by up to 50%. This shows that intensification of land use plays a key role when addressing competing land claims. Impacts may be underestimated if wood production is ignored and forests are considered as homogenous natural habitat.

### What Difference Does it Make?

We found that, even though future forest cover changes will have the largest impact on biodiversity, changes in wood production patterns will also significantly affect biodiversity. While global average impacts are rather small, effects vary for different global regions and taxa, and for some the difference may be substantial. Therefore, careful interpretation of global scale indicators is necessary, as regional and local impacts may be much more pronounced. While the impacts of wood production might seem small, neglecting them will always result in an underestimation of the impacts of forest use on biodiversity.

We found generally larger wood production impacts in regions in the global North, compared to the South. On the one hand, this is caused by the adopted species richness values from Chaudhary et al. (2016). Furthermore, the global North has less deforestation, resulting in larger differences between accounting for wood production or not. For most taxa-region combinations, the SSP1 scenario had the lowest negative or the highest positive impact on species richness and showed a lower decrease of HSR. This emphasizes the need and benefits of striving toward more sustainable resource use.

Our estimations on the impacts on biodiversity are rather optimistic and could be considered an underestimation for several reasons. First, only species restricted to forest were considered. Species that occur in other habitats, but depend on forests, e.g. for breeding or feeding, were ignored. Second, due to data availability, only amphibian, bird, mammal and reptile species were included. Plant species biodiversity, for example, can decrease considerably due to changes in microclimate and habitat fragmentation through forest plantations (Braun et al. 2017; Bremer and Farley 2010; Farmilo et al. 2013). Third, we did not consider interconnected impacts and consequences of wood harvest and forest plantations, such as disturbed natural forest fire regimes, changes in water quality and quantity or exotic species, pests and pathogens (for example Ennos et al. 2019; Hess and Tschinkel 2017; Scott and Gush 2017).

### Limitations

We used the most recent and most detailed data on current and future forest management. Nevertheless, there are large uncertainties related to the spatial data used (Schulze et al. 2019; van Asselen and Verburg 2012). Different forest definitions in different source data could have led to misrepresentations of areas that are important for biodiversity and/or wood production. The recently developed Spatial Database of Planted Trees (Harris et al. 2019) likely outperforms the maps of forest classes and uses in terms of accuracy of the location of planted forests. However, the gain in accuracy comes with losses in temporal and spatial consistency (Harris et al. 2019), which is necessary for global analysis as conducted here. The likelihood maps adopted from Schulze et al. (2019) are subject to uncertainties due to potentially biased data and the fact that they are entirely based on current conditions. National and regional data on wood harvest remains sparse and different reporting schemes and classifications create uncertainties (Erb et al. 2017; Schulze et al. 2019).

We used estimates from the scientific literature for most of the parameters applied in the calculations. For example, current shares and future changes in plantation wood are estimates provided by national experts, collected by d’Annunzio et al. (2015) and Del Lungo et al. (2006). In some instances, however, we had to make own assumptions, since data from the literature were not available (listed in ESM section 1.5 and 1.8). For example, the share of roundwood utilizable from deforestation wood is based on our own assumption. All these assumptions come with considerable uncertainties that are common in this type of studies, but should be accounted for in interpreting the results. Confidence bands were not available for the data used on wood consumption or the projections of future forest extents. Therefore, it is not possible to give an uncertainty assessment of our results.

Additional uncertainties are related to estimated wood available from deforestation. The data on forest cover loss also cover areas that are not converted but clear-cut for timber harvest (Curtis et al. 2018). Double counting of those areas was avoided by solely including loss areas outside the forest extent. While it is not clear, to which extent the amount of wood from deforestation accounts toward official roundwood production statistics (Hurtt et al. 2011), a clear link between timber production and deforestation has been found (Damette and Delacote 2011). More research is necessary to determine the amount of wood derived from deforestation with higher accuracy, as it would be false to ignore it entirely. Wood in burnt areas was excluded completely, which is likely an overestimation, since post-fire salvage logging or harvest of timber before land conversion often occurs. The applied dataset on burnt areas (Giglio et al. 2013) addresses the challenges of detecting forest fires in closed canopy forests and estimating the extent of deforestation fires by incorporating data on tree cover, fire persistence and estimates from field observations (Giglio et al. 2006).

For all scenarios, the same increase of the share in plantation wood, the increase in plantation productivity, and the combination of forest management types were applied. Ideally, these three parameters should be an integral, variable, part of the storylines of the SSP scenarios. For example, the SSP1 scenario could have a higher share of more sustainable harvest practices in non-planted forests, such as reduced impact logging or selection cut. Furthermore, mitigation strategies can also change the composition of forest and management types as a simulation of forests in Europe has shown (Luyssaert et al. 2018). Impacts of climate change were not included in our study, which are expected to affect forest productivity, water availability as well as species composition and response (Medlyn et al. 2011; Morin et al. 2018). Furthermore, climate change will likely cause a shift in wood harvest suitability and species ranges. The biodiversity indicators, which we investigated, are commonly used in global assessments (Jetz et al. 2007; Kehoe et al. 2017; Newbold et al. 2016; Powers and Jetz 2019). They, however, do not cover the whole complexity of biodiversity. Other indicators that would be interesting to consider, include species intactness indices or multivariate community intactness indices (Lamb et al. 2009). The data on forest management impact on species richness response came from a comprehensive review study (Chaudhary et al. 2016). For taxa-management combinations that were not available for a region, a global average was used. This procedure includes major uncertainties. Furthermore, we could not differentiate between different types of plantations, which can be very different in different regions of the world. Because of that, the species response for non-planted wood production was lower than for planted wood production for mammals in Africa and South America (Table S_2). This is rather unlikely, since wood plantations usually have a larger impact on biodiversity than logged natural forests (Brockerhoff et al. 2008). Furthermore, a distinction between native and non-native planted forests was not possible. Also for non-planted wood production there are uncertainties remaining, even though more detailed data were available. Within one region, they can be managed very differently, resulting in different impacts on forest properties and biodiversity (Schelhaas et al. 2018). Species richness responses for single species in different areas would be desirable, but those are (currently) not available on a global scale. The PREDICTS database provides a big step toward providing better empirical basis for biodiversity assessments (Hudson et al. 2017). However, data on the effects of different forest use types remains underrepresented in this database. The PREDICTS database does include plantation forests; however, this category also includes crop trees, such as oil palm or fruit trees. Harvested forests are not included as a separate category, but different age levels can be indicative for wood harvest, as a recent study has shown (Phillips et al. 2017). We furthermore considered new forests to support species richness to the same degree as existing ones, which is likely an overestimation as restored forests often cannot support biodiversity to the same extent (Chazdon 2008).

While this study is limited by the uncertainties of the variety of data used, we have shown that changes in forest use systems have significant impacts on biodiversity. Therefore, we emphasize that more detailed information on forest use and type should be included in land change studies. Their inclusion could support policies on biodiversity protection, international afforestation commitments, and studies on degradation, restoration or climate change.

## Supplementary information

Supplementary Information
