# Supplementary material for: The Impact of Accounting for Future Wood Production in Global Vertebrate Biodiversity Assessments
Source: Environ Manage. 2020 Jul 5;66(3):460–75. doi: 10.1007/s00267-020-01322-4 (PMC7434756; doi:10.1007/s00267-020-01322-4)
Supplement: Supplementary file 1 — Supplementary Information [file 267_2020_1322_MOESM1_ESM.docx]

Electronic supplementary material

Schulze et al.: Biodiversity impacts of future wood production

# Methodology

## Forest extent in 2000

The forest cover of the land system map (van Asselen and Verburg 2012) was resampled from a 9.25 x 9.25 km^2^ resolution to 1 x 1 km^2^, to match with the maps of forest classes and uses (Schulze et al. 2019). When overlaying the two maps, 100% forest cover was assigned to dense and open forests and 50% to mosaic forest systems. Since the forest class and use maps are limited to reported statistics and the land system map is not, the forest extent of both maps differs. Only forest areas occurring in both classifications were considered for further analysis (Figure S_1 and S_2).


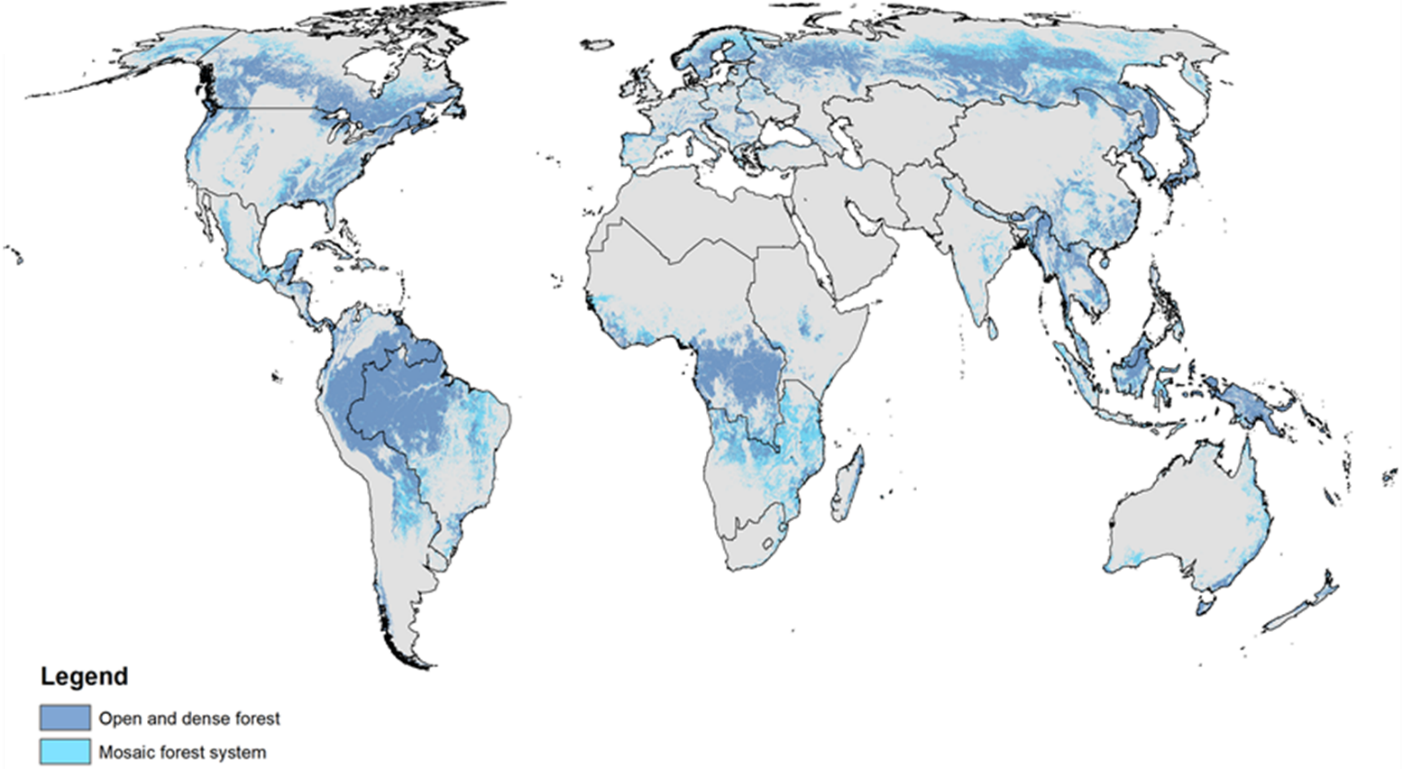


Fig. S_1 *Forest extent and cover in the year 2000. We assumed 100% forest cover for open and dense forest and 50% for mosaic forest systems*

## Forest classes and uses in 2000

| **Tab. S_1** *Area of non-productive forests, non-planted and planted wood production in 2000* | | | |
| --- | --- | --- | --- |
|  |  | Area in 2000 [km^2^] |  |
| Region | Non-productive forest | Non-planted wood production | Planted wood production |
| North America | 4,454,314 | 565,641 | 260,804 |
| South and Central America | 7,374,881 | 451,610 | 68,024 |
| Europe and Turkey | 433,940 | 460,578 | 314,856 |
| Africa | 3,088,290 | 1,172,915 | 30,181 |
| Northern, Central, Western Asia | 2,661,165 | 2,900,584 | 77,370 |
| South and South-East Asia | 1,760,881 | 643,343 | 71,770 |
| Eastern Asia and Oceania | 1,543,451 | 253,873 | 281,899 |


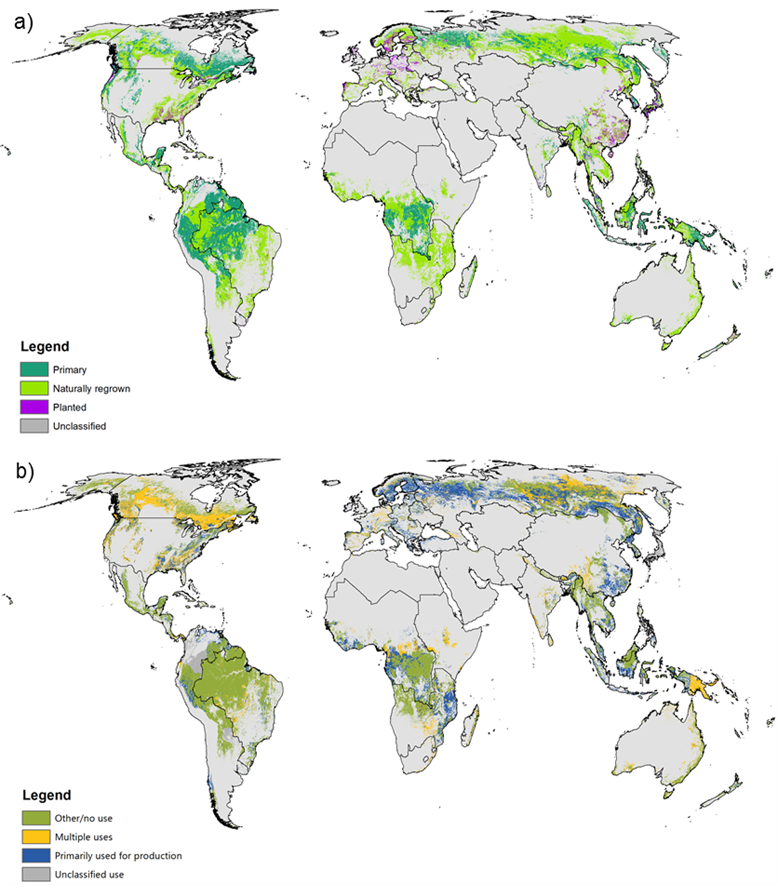


Fig. S_2 *Adapted forest classes and uses from the maps by Schulze et al. (2019). Only forest areas within the forest cover of the land system map by van Asselen and Verburg (2012) were considered*

## List of countries included in this study, grouped by major region (bold) and region (italic)

**North America**

- *Canada*
- *USA*
- *Mexico*

**Central and South America**

- *Central America*
  - Antigua and Barbuda
  - Bahamas
  - Barbados
  - Belize
  - Bermuda
  - Costa Rica
  - Cuba
  - Dominica
  - Dominican Republic
  - El Salvador
  - Grenada
  - Guadeloupe
  - Guatemala
  - Haiti
  - Honduras
  - Jamaica
  - Martinique
  - Nicaragua
  - Panama
  - Puerto Rico
  - Saint Vincent and Grenadines
  - Trinidad and Tobago
- *Brazil*
  - Brazil
- *Rest of South America*
  - Argentina
  - Bolivia
  - Chile
  - Colombia
  - Ecuador
  - Guyana
  - Paraguay
  - Peru
  - Suriname
  - Uruguay
  - Venezuela

**Africa**

- *Northern Africa*
  - Algeria
  - Egypt
  - Libya
  - Morocco
  - Tunisia
- *Western Africa*
  - Benin
  - Burkina Faso
  - Cameroon
  - Central African Republic
  - Chad
  - Cote d'Ivoire
  - Democratic Republic of the Congo
  - Equatorial Guinea
  - Gabon
  - Gambia
  - Ghana
  - Guinea
  - Guinea-Bissau
  - Liberia
  - Mali
  - Niger
  - Nigeria
  - Republic of the Congo
  - Sao Tome and Principe
  - Senegal
  - Sierra Leone
  - Togo
- *Eastern Africa*
  - Burundi
  - Comoros
  - Ethiopia
  - Kenya
  - Madagascar
  - Mauritius
  - Reunion
  - Rwanda
  - Somalia
  - South Sudan
  - Sudan
  - Uganda
- *Rest of Southern Africa*
  - Angola
  - Botswana
  - Lesotho
  - Malawi
  - Mozambique
  - Namibia
  - Zimbabwe
  - Swaziland
  - Tanzania
  - Zambia
- *South Africa*

**South and South-East Asia**

- *Southeastern Asia*
  - Brunei Darussalam
  - Cambodia
  - Lao PDR
  - Malaysia
  - Myanmar
  - Philippines
  - Thailand
  - Viet Nam
- *Indonesia region*
  - Indonesia
  - Papua New Guinea
  - Timor-Leste
- *India*
- *Rest of South Asia*
  - Afghanistan
  - Bangladesh
  - Bhutan
  - Nepal
  - Pakistan
  - Sri Lanka

**East Asia and Oceania**

- *Korea region*
  - Democratic People's Republic of Korea
  - Republic of Korea
- *China region*
  - China
  - Mongolia
- *Japan*
- *Oceania*
  - Australia
  - Fiji
  - New Caledonia
  - New Zealand
  - Solomon Islands
  - Vanuatu

**Northern, Central and Western, Asia**

- *Central Asia*
  - Kazakhstan
  - Kyrgyz Republic
  - Tajikistan
  - Uzbekistan
- *Russia region*
  - Armenia
  - Azerbaijan
  - Georgia
  - Russian Federation
- *Middle East*
  - Islamic Republic of Iran
  - Iraq
  - Israel
  - Lebanon
  - Syrian Arab Republic

**Europe and Turkey**

- *Western Europe*
  - Andorra
  - Austria
  - Belgium
  - Denmark
  - Finland
  - France
  - Germany
  - Greece
  - Iceland
  - Ireland
  - Italy
  - Luxembourg
  - Netherlands
  - Norway
  - Portugal
  - Spain
  - Sweden
  - Switzerland
  - United Kingdom
- *Central Europe*
  - Albania
  - Bosnia and Herzegovina
  - Bulgaria
  - Croatia
  - Cyprus
  - Czech Republic
  - Estonia
  - Hungary
  - Latvia
  - Lithuania
  - Macedonia
  - Poland
  - Romania
  - Serbia and Montenegro
  - Slovakia
  - Slovenia
- *Turkey*
- *Ukraine region*
  - Belarus
  - Moldova
  - Ukraine

## Relative changes of timber supplies in 2050 following 3 SSPs, relative to 2000


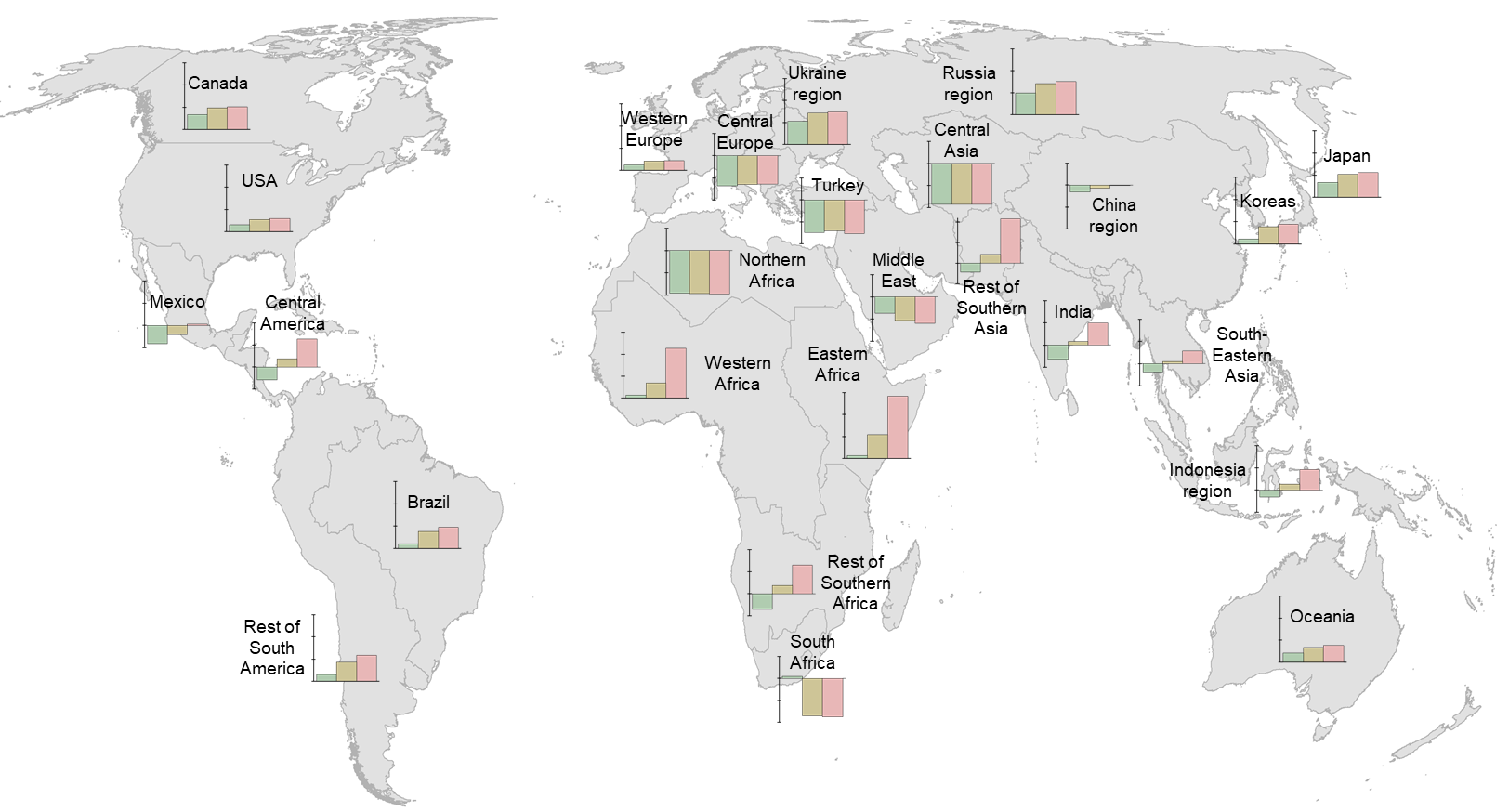

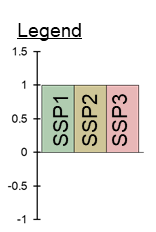


Fig. S_3 *Relative change in timber supplies. Each tick mark represents 0.5, i.e. 50% change. The original data sources of timber supply in 2005 and 2050 are Doelman et al. (2018) and van Vuuren et al. (2017)*

## Roundwood that can be used from deforestation wood

Industrial roundwood:

- Boreal & temperate forests: 75% (own estimate)
- (Sub-) Tropical forests: 15% (Arets et al. 2011)

Wood fuel:

- 95% in all regions (own estimate)

Conversion factor biomass to roundwood:

- 1.3 in all regions (Arets et al. 2011).

## List of countries without forest designated primarily for production (following Schulze et al. 2019 and Food and Agriculture Organization of the United Nations 2016)

| - Afghanistan | - Germany | - Namibia |
| --- | --- | --- |
| - Armenia | - Israel | - Niger |
| - Belgium | - Japan | - Singapore |
| - Botswana | - Kazakhstan | - Syrian Arab Republic |
| - Burkina Faso | - Mali | - United Arab Emirates |
| - Djibouti | - Moldova | - Yemen |

In these countries, we assumed that 30% of multiple use forests were used for wood production.

## Likelihood maps

To create an updated plantation likelihood map in regions with dense national or subnational coverage, 1000 points were randomly sampled within the mapped planted forest areas. These were used as the dependent variable in a logistic regression model. The selection of independent variables was adapted from Schulze et al. (2019) and is listed and briefly described in Table S_2. The choice of predictor variables built on findings of previous studies and spatial availability on a global scale. Model selection was performed stepwise on basis of the lowest Akaike information criterion value.

| **Tab. S_2** *Predictor variables for modelling likelihoods for the occurrence of plantation and production forests (from Schulze et al. 2019)* | | | |
| --- | --- | --- | --- |
| Predictor | Description | Unit | Source |
| NPP | Net Primary Productivity | Units of elemental C | Imhoff et al. 2004 |
| Tree density | Percentage tree cover | % | ISCGM et al. 2008 |
| Forest loss | Forest change between 2000 – 2014, aggregated with summation | binary | Hansen et al. 2013 |
| Forest gain | Forest change between 2000 – 2014, aggregated with summation | binary | Hansen et al. 2013 |
| Elevation | Altitude in meters above average sea level | m | GTOPO30:  USGS EROS 1996 |
| Slope | Derived from elevation map | % | Own calculation |
| Sand content | Share of sand in soil texture | % | S-World:  Stoorvogel et al. 2017 |
| Clay content | Share of clay in soil texture | % | S-World:  Stoorvogel et al. 2017 |
| Soil depth | Total depth of the soil | cm | S-World:  Stoorvogel et al. 2017 |
| Depth of topsoil | Depth of the topsoil layer | cm | S-World:  Stoorvogel et al. 2017 |
| C content in subsoil | Carbon content in the subsoil layer | % | S-World:  Stoorvogel et al. 2017 |
| C content in top 50 cm | Carbon content in the layers of the top 50 cm of the soil | % | S-World:  Stoorvogel et al. 2017 |
| C content in topsoil | Carbon content in the topsoil layer | % | S-World:  Stoorvogel et al. 2017 |
| Drainage classes | Soil drainage differentiating between 7 different classes from 1: Very poorly drained to 7: Excessively drained | Categorical | WISE30:  Hengl et al. 2014 |
| Aridity Index | Ratio between average yearly precipitation and average yearly potential evapotranspiration. | No unit | CGIAR-CSI Global-Aridity and Global-PET Database:  Zomer et al. 2007; Zomer et al. 2008 |
| Market accessibility | Distance and travel time to international and regional markets | No unit | Verburg et al. 2011 |
| Travel time to major cities | Accessibility measured in travel time to cities with >50.000 inhabitants | hours | Uchida & Nelson 2009; Uchida & Nelson 2010 |
| Distance to roads | Euclidian distance to roads calculated based on the most consistent global road map | km | Dubinin 2014 |
| Distance to rivers | Euclidian distance to rivers | km | Pape 2004 |
| Population density 2015 | Population density, downscaled with urban built-up areas | People/km^2^ | Freire & Pesaresi 2015 |
| Aggregated governance index | Determines perception of governance within a country. Aggregation by sum of 4 indices: regulatory quality, government effectiveness, control of corruption and rule of law | No unit | Kaufmann & Kraay 2017 |

## Productive plantations

To distinguish plantations used for wood production from those that are used for other purposes (e.g. control of erosion or desertification, restoration), we used the shares given in Del Lungo et al. (2006). Those were partially modified, as follows:

1. If the Del Lungo dataset did not report any planted forests, we used the share of planted semi-natural forest area, instead.
2. If the area of production and plantation forests was exactly the same, we assumed that 100% of plantations are productive

**Fig. S_4** *Distribution of productive and non-productive plantation forests. The shares of productive plantations by country were adapted from Del Lungo et al. (2006). They were allocated within the plantation extent (Figure S_2) with help of likelihood maps for forests primarily used for production (for more information see above - section 1.8). They can be accessed at* [*www.environmentalgeography.nl*](http://www.environmentalgeography.nl) *(upon publication)*


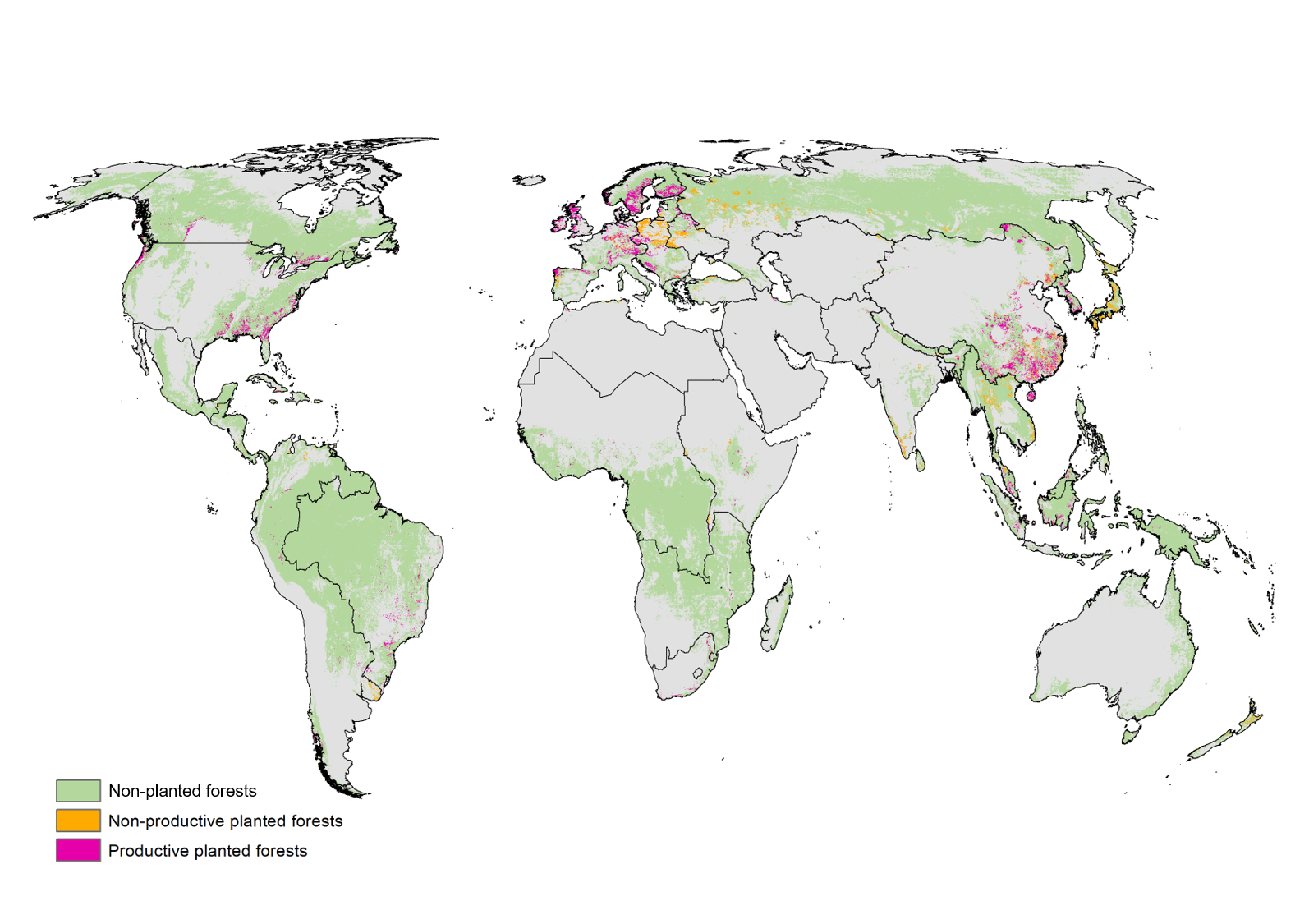


## Species response values

We used average species response values from Chaudhary et al. (2016). If available, we used separate values for each taxa-region-management combination. In many cases, however, we had to rely on global values. If also no global value was given, we applied a species response of 1 (i.e. no change). Species response for production forests were weighted with the respective area of harvest type (e.g. clear-cut, selective cut, shelter wood).

| **Tab. S_3a** *Species response values for planted production forests as applied in this study, adopted from Chaudhary et al. (2016). For planted production forests we averaged (Av.) the species response values for timber and fuel plantations. If species response was missing for a taxa-continent combination, the global average was applied (italic). A value of 1 is equal to no change* | | | | | | | | | | |
| --- | --- | --- | --- | --- | --- | --- | --- | --- | --- | --- |
|  | **Amphibians** | | | **Birds** | | | **Mammals** | | |  |
|  | Timber | Fuel | **Av.** | Timber | Fuel | **Av.** | Timber | Fuel | **Av.** |  |
| North America | *0.833* | *0.277* | **0.555** | *0.637* | *0.398* | **0.517** | *1.100* | *0.705* | **0.903** |  |
| South and Central America | *0.833* | *0.277* | **0.555** | *0.637* | 0.369 | **0.503** | *1.100* | *0.705* | **0.903** |  |
| Europe and Turkey | *0.833* | *0.277* | **0.555** | *0.637* | *0.398* | **0.517** | *1.100* | *0.705* | **0.903** |  |
| Africa | *0.833* | *0.277* | **0.555** | 0.818 | *0.398* | **0.608** | *1.100* | *0.705* | **0.903** |  |
| North, Central, Western Asia | *0.833* | *0.277* | **0.555** | *0.637* | *0.398* | **0.517** | *1.100* | *0.705* | **0.903** |  |
| South and South-East Asia | *0.833* | *0.277* | **0.555** | *0.637* | *0.398* | **0.517** | *1.100* | *0.705* | **0.903** |  |
| East Asia and Oceania | *0.833* | *0.277* | **0.555** | *0.637* | *0.398* | **0.517** | *1.100* | *0.705* | **0.903** |  |

| **Tab. S_3b** *Species response values for non-planted production forests as applied in this study for amphibians (A), birds (B) and mammals (M), adopted from Chaudhary et al. (2016). The values were weighed by the share (S) of forest management practices within each region using estimations from Arets et al. (2011) and then averaged for the major regions (see section 1.4, final results in Table S_3c). For temperate and boreal countries, clear-cut, selection system and retention were weighed equally since data on the share was not available. If species response was missing for a taxa-continent combination, the global average was applied (italic). A value of 1 is equal to no change* | | | | | | | | | | | | | | | | | | | | | | |
| --- | --- | --- | --- | --- | --- | --- | --- | --- | --- | --- | --- | --- | --- | --- | --- | --- | --- | --- | --- | --- | --- | --- |
|  | Clear-cut | | | | Selection system | | | | Retention | | | | Selective logging | | | | Reduced impact logging | | | | |  |
|  | A | B | M | S | A | B | M | S | A | B | M | S | A | B | M | S | A | B | M | S | |  |
| Canada | 0.54 | 0.61 | 1.39 | 0.33 | *1.00* | *1.25* | *1.00* | 0.33 | *1.00* | 1.00 | 1.42 | 0.33 |  |  |  |  |  |  |  |  | |  |
| USA | 0.54 | 0.61 | 1.39 | 0.33 | *1.00* | *1.25* | *1.00* | 0.33 | *1.00* | 1.00 | 1.42 | 0.33 |  |  |  |  |  |  |  |  | |  |
| Mexico |  |  |  |  |  |  |  |  |  |  |  |  | *1.07* | *0.83* | *0.83* | 0.84 | *1.37* | *1.00* | *0.81* | 0.16 | |  |
|  |  |  |  |  |  |  |  |  |  |  |  |  |  |  |  |  |  |  |  |  | |  |
| Central America |  |  |  |  |  |  |  |  |  |  |  |  | *1.07* | 0.83 | *0.83* | 0.86 | *1.37* | 0.97 | *0.81* | 0.14 | |  |
| Brazil | *0.54* | *0.59* | *1.39* | 0.11 |  |  |  |  |  |  |  |  | *1.07* | 0.83 | *0.83* | 0.62 | *1.37* | 0.97 | *0.81* | 0.27 | |  |
| Rest of South America | *0.54* | *0.59* | *1.39* | 0.02 |  |  |  |  |  |  |  |  | *1.07* | 0.83 | *0.83* | 0.87 | *1.37* | 0.97 | *0.81* | 0.11 | |  |
|  |  |  |  |  |  |  |  |  |  |  |  |  |  |  |  |  |  |  |  |  | |  |
| Western Europe | *0.54* | *0.59* | *1.39* | 0.33 | *1.00* | *1.25* | *1.00* | 0.33 | *1.00* | *1.00* | *1.42* | 0.33 |  |  |  |  |  |  |  |  | |  |
| Central Europe | *0.54* | *0.59* | *1.39* | 0.33 | *1.00* | *1.25* | *1.00* | 0.33 | *1.00* | *1.00* | *1.42* | 0.33 |  |  |  |  |  |  |  |  | |  |
| Ukraine region | *0.54* | *0.59* | *1.39* | 0.33 | *1.00* | *1.25* | *1.00* | 0.33 | *1.00* | *1.00* | *1.42* | 0.33 |  |  |  |  |  |  |  |  | |  |
| Turkey | *0.54* | *0.59* | *1.39* | 0.33 | *1.00* | *1.25* | *1.00* | 0.33 | *1.00* | *1.00* | *1.42* | 0.33 |  |  |  |  |  |  |  |  | |  |
| **Tab. S_3b (cont.)** | | | | | | | | | | | | | | | | | | | | | |  |
|  | Clear-cut | | | | Selection system | | | | Retention | | | | Selective logging | | | | Reduced impact logging | | | | |  |
|  | A | B | M | S | A | B | M | S | A | B | M | S | A | B | M | S | A | B | M | S | |  |
| Northern Africa |  |  |  |  |  |  |  |  |  |  |  |  | 1.05 | *0.83* | *0.83* | 1.00 |  |  |  |  | |  |
| Western Africa |  |  |  |  |  |  |  |  |  |  |  |  | 1.05 | *0.83* | *0.83* | 0.98 | *1.37* | *1.00* | *0.81* | 0.02 | |  |
| Eastern Africa |  |  |  |  |  |  |  |  |  |  |  |  | 1.05 | *0.83* | *0.83* | 0.99 | *1.37* | *1.00* | *0.81* | 0.01 | |  |
|  |  |  |  |  |  |  |  |  |  |  |  |  |  |  |  |  |  |  |  |  | |  |
| Russia region | *0.54* | *0.59* | *1.39* | 0.33 | *1.00* | *1.25* | *1.00* | 0.33 | *1.00* | *1.00* | *1.42* | 0.33 |  |  |  |  |  |  |  |  | |  |
| Central Asia | *0.54* | *0.59* | *1.39* | 0.33 | *1.00* | *1.25* | *1.00* | 0.33 | *1.00* | *1.00* | *1.42* | 0.33 |  |  |  |  |  |  |  |  | |  |
|  |  |  |  |  |  |  |  |  |  |  |  |  |  |  |  |  |  |  |  |  |  |  |
| Southeast Asia | *0.54* | *0.59* | *1.39* | 0.43 |  |  |  |  |  |  |  |  | *1.07* | 0.83 | 0.78 | 0.34 | *1.37* | *1.00* | *0.81* | 0.22 | |  |
| Indonesia region | *0.54* | *0.59* | *1.39* | 0.30 |  |  |  |  |  |  |  |  | *1.07* | 0.83 | 0.78 | 0.63 |  |  |  |  | |  |
| India | *0.54* | *0.59* | *1.39* | 0.63 |  |  |  |  |  |  |  |  | *1.07* | 0.83 | 0.78 | 0.37 |  |  |  |  | |  |
|  |  |  |  |  |  |  |  |  |  |  |  |  |  |  |  |  |  |  |  |  | |  |
| China region | *0.54* | *0.59* | *1.39* | 0.33 | *1.00* | *1.25* | *1.00* | 0.33 | *1.00* | *1.00* | *1.42* | 0.33 |  |  |  |  |  |  |  |  | |  |
| Korea region | *0.54* | *0.59* | *1.39* | 0.33 | *1.00* | *1.25* | *1.00* | 0.33 | *1.00* | *1.00* | *1.42* | 0.33 |  |  |  |  |  |  |  |  | |  |
| Japan | *0.54* | *0.59* | *1.39* | 0.33 | *1.00* | *1.25* | *1.00* | 0.33 | *1.00* | *1.00* | *1.42* | 0.33 |  |  |  |  |  |  |  |  | |  |
| Oceania | *0.54* | *0.59* | *1.39* | 0.33 | *1.00* | *1.25* | *1.00* | 0.33 | *1.00* | *1.00* | *1.42* | 0.33 |  |  |  |  |  |  |  |  | |  |
|  |  |  |  |  |  |  |  |  |  |  |  |  |  |  |  |  |  |  |  |  | |  |

| **Tab. S_3c** *Species response values as applied in this study. Comprehensive data per wood harvest type can be found in Table 2a & b. A value of 1 is equal to no change* | | | | | | | | |
| --- | --- | --- | --- | --- | --- | --- | --- | --- |
|  | **Amphibians** | | | **Birds** | | | **Mammals** | |
|  | Non-planted | Planted | Non-planted | | Planted | Non-planted | | Planted |
| North America | 0.737 | 0.555 | 0.921 | | 0.517 | 1.212 | | 0.903 |
| South and Central America | 1.101 | 0.555 | 0.847 | | 0.507 | 0.849 | | 0.903 |
| Europe and Turkey | 0.544 | 0.555 | 0.947 | | 0.517 | 1.406 | | 0.903 |
| Africa | 1.035 | 0.555 | 0.899 | | 0.608 | 0.897 | | 0.903 |
| North, Central, Western Asia | 0.696 | 0.555 | 0.965 | | 0.517 | 1.270 | | 0.903 |
| South and South-East Asia | 0.831 | 0.555 | 0.724 | | 0.517 | 1.086 | | 0.903 |
| East Asia and Oceania | 0.750 | 0.555 | 0.959 | | 0.517 | 1.257 | | 0.903 |

# Results

**Fig. S_5** *Relative changes in planted and non-planted timber production area by region, following 3 SSP scenarios*


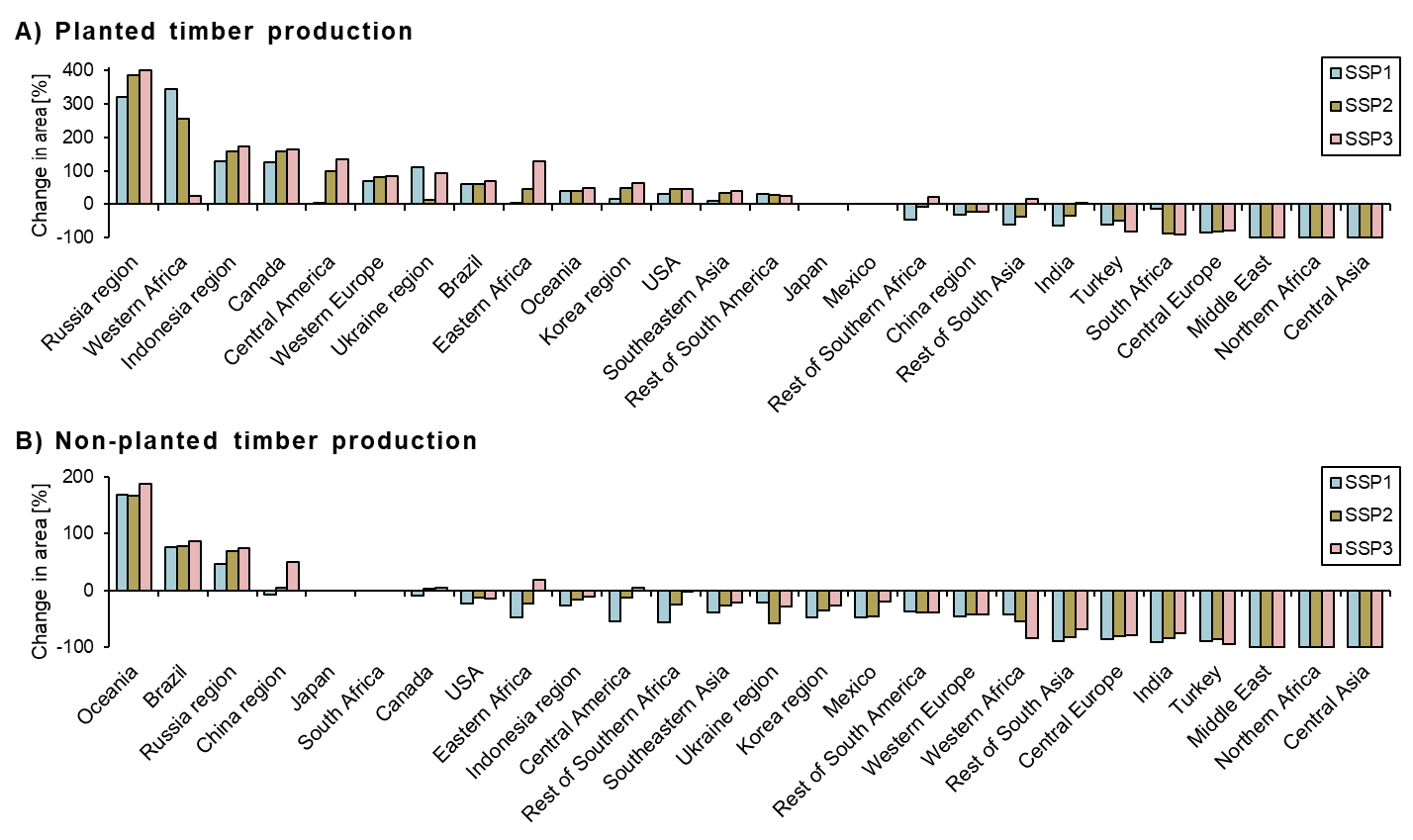


| **Tab. S_4** *Timber yield and production forest area in 2000 and estimated area and relative change in 2050 following 3 SSP projections for timber demand.* | | | | | | | | | | | | | | | | | |
| --- | --- | --- | --- | --- | --- | --- | --- | --- | --- | --- | --- | --- | --- | --- | --- | --- | --- |
| **Region** | **Yield [‘000 m3/km^2^]** | | | **Area 2000 [km^2^]** | | **Area 2050 in SSP1 scenario [km^2^]** | | | | **Area 2050 in SSP2 scenario [km^2^]** | | | | **Area 2050 in SSP3 scenario [km^2^]** | | | |
|  | Planted | | Non-planted | Planted | Non-planted | Planted | | Non-planted | | Planted | | Non-planted | | Planted | | Non-planted | |
|  | 2000 | 2050 |  |  |  | Absolute | %Change | Absolute | %Change | Absolute | %Change | Absolute | %Change | Absolute | %Change | Absolute | %Change |
| **Canada** | 0.56 | 0.66 | 1.24 | 76,753 | 92,329 | 173,047 | 125 | 83,031 | -10 | 198,977 | 159 | 95,473 | 3 | 201,919 | 163 | 96,885 | 5 |
| **USA** | 0.87 | 1.29 | 0.65 | 184,051 | 444,073 | 237,153 | 29 | 341,271 | -23 | 267,847 | 46 | 385,434 | -13 | 265,417 | 44 | 382,027 | -14 |
| **Mexico** | 0.00 | 0.00 | 0.53 | 0 | 29,239 | 0 | 0 | 15,182 | -48 | 0 | 0 | 15,951 | -45 | 0 | 0 | 23,398 | -20 |
|  |  |  |  |  |  |  |  |  |  |  |  |  |  |  |  |  |  |
| **Central America** | 2.53 | 3.22 | 0.55 | 2,688 | 15,539 | 2,738 | 2 | 6,984 | -55 | 5,330 | 98 | 13,594 | -13 | 6,292 | 134 | 16,272 | 5 |
| **Brazil** | 2.39 | 2.96 | 0.11 | 27,917 | 106,025 | 44,658 | 60 | 187,424 | 77 | 44,940 | 61 | 188,613 | 78 | 46,934 | 68 | 196,978 | 86 |
| **Rest of South America** | 1.19 | 2.36 | 0.03 | 37,419 | 330,046 | 48,300 | 29 | 208,770 | -37 | 47,116 | 26 | 203,637 | -38 | 46,399 | 24 | 200,511 | -39 |
|  |  |  |  |  |  |  |  |  |  |  |  |  |  |  |  |  |  |
| **Western Europe** | 0.59 | 0.70 | 0.60 | 222,605 | 299,280 | 375,275 | 69 | 159,955 | -47 | 401,847 | 81 | 171,292 | -43 | 405,653 | 82 | 173,021 | -42 |
| **Central Europe** | 1.23 | 1.56 | 0.40 | 65,814 | 102,809 | 9,192 | -86 | 14,256 | -86 | 12,551 | -81 | 19,472 | -81 | 13,223 | -80 | 20,481 | -80 |
| **Ukraine region** | 0.22 | 0.28 | 0.21 | 16,624 | 29,919 | 35,203 | 112 | 23,390 | -22 | 18,486 | 11 | 12,281 | -59 | 31,859 | 92 | 21,155 | -29 |
| **Turkey** | 0.09 | 0.13 | 0.48 | 9,813 | 28,570 | 3,821 | -61 | 3,139 | -89 | 4,831 | -51 | 3,965 | -86 | 1,618 | -84 | 1,325 | -95 |
|  |  |  |  |  |  |  |  |  |  |  |  |  |  |  |  |  |  |
| **Northern Africa** | 0.02 | 0.02 | 0.01 | 786 | 1,332 | 0 | -100 | 0 | -100 | 0 | -100 | 0 | -100 | 0 | -100 | 0 | -100 |
| **Western Africa** | 1.29 | 3.69 | 0.09 | 5,421 | 766,378 | 24,183 | 346 | 436,131 | -43 | 19,262 | 255 | 347,378 | -55 | 6,784 | 25 | 122,269 | -84 |
| **Eastern Africa** | 4.15 | 4.57 | 0.44 | 9,579 | 25,562 | 9,736 | 2 | 13,556 | -47 | 13,996 | 46 | 19,487 | -24 | 21,754 | 127 | 30,257 | 18 |
| **South Africa** | 2.45 | 2.89 | 0.00 | 9,622 | 0 | 8,237 | -14 | 0 | 0 | 1,018 | -89 | 0 | 0 | 775 | -92 | 0 | 0 |
| **Rest of Southern Africa** | 1.07 | 2.11 | 0.07 | 4,773 | 379,643 | 2,544 | -47 | 163,607 | -57 | 4,368 | -8 | 280,987 | -26 | 5,789 | 21 | 372,326 | -2 |
|  |  |  |  |  |  |  |  |  |  |  |  |  |  |  |  |  |  |
| **Russia region** | 0.31 | 0.36 | 0.02 | 75,587 | 2,900,543 | 317,904 | 321 | 4,240,161 | 46 | 368,505 | 388 | 4,915,025 | 69 | 379,251 | 402 | 5,058,387 | 74 |
| **Central Asia** | 10.01 | 19.82 | 87.40 | 20 | 1 | 0 | -100 | 0 | -100 | 0 | -100 | 0 | -100 | 0 | -100 | 0 | -100 |
| **Middle East** | 0.01 | 0.01 | 0.81 | 1,763 | 40 | 0 | -100 | 0 | -100 | 0 | -100 | 0 | -100 | 0 | -100 | 0 | -100 |
|  |  |  |  |  |  |  |  |  |  |  |  |  |  |  |  |  |  |
| **Southeast Asia** | 0.78 | 0.96 | 0.19 | 43,193 | 187,880 | 47,060 | 9 | 114,088 | -39 | 57,118 | 32 | 138,468 | -26 | 60,413 | 40 | 147,226 | -22 |
| **Indonesia region** | 0.52 | 0.63 | 0.05 | 19,074 | 389,329 | 43,298 | 127 | 287,337 | -26 | 49,420 | 159 | 327,962 | -16 | 51,736 | 171 | 346,200 | -11 |
| **India** | 7.91 | 11.38 | 0.21 | 6,045 | 62,594 | 2,226 | -63 | 5,283 | -92 | 3,979 | -34 | 9,450 | -85 | 6,308 | 4 | 15,069 | -76 |
| **Rest of South Asia** | 4.66 | 6.71 | 1.04 | 3,458 | 3,540 | 1,311 | -62 | 357 | -90 | 2,184 | -37 | 595 | -83 | 4,043 | 17 | 1,094 | -69 |
|  |  |  |  |  |  |  |  |  |  |  |  |  |  |  |  |  |  |
| **China region** | 0.48 | 0.54 | 0.09 | 240,377 | 195,757 | 162,164 | -33 | 180,383 | -8 | 182,683 | -24 | 203,199 | 4 | 188,117 | -22 | 294,453 | 50 |
| **Korea region** | 0.15 | 0.21 | 0.05 | 17,164 | 53,744 | 19,992 | 16 | 27,812 | -48 | 25,185 | 47 | 35,042 | -35 | 27,817 | 62 | 38,927 | -28 |
| **Japan** | 0.00 | 0.00 | 0.00 | 0 | 0 | 0 | 0 | 0 | 0 | 0 | 0 | 0 | 0 | 0 | 0 | 0 | 0 |
| **Oceania** | 1.53 | 1.71 | 0.08 | 24,358 | 4,372 | 34,061 | 40 | 11,771 | 169 | 33,798 | 39 | 11,685 | 167 | 36,398 | 49 | 12,587 | 188 |

| **Tab. S_5:** *Aggregated species richness values, divided by 1000 and expressed in mean ratio, for amphibians, birds, mammals and the three taxa combined for the year 2000 and for 2050, following three SSP scenarios* | | | | | | | | | |
| --- | --- | --- | --- | --- | --- | --- | --- | --- | --- |
|  |  | Forest cover change | | | | Forest cover & wood production change | | | |
|  | Region | 2000 | 2050 - SSP1 | 2050 - SSP2 | 2050 - SSP3 | 2000 | 2050 - SSP1 | 2050 - SSP2 | 2050 - SSP3 |
| Amphibians | Global | 36,486 | 36,088 | 32,920 | 31,812 | 36,468 | 36,002 | 32,914 | 31,783 |
|  | North America | 822 | 737 | 624 | 647 | 756 | 664 | 542 | 559 |
|  | South and Central America | 29,543 | 29,247 | 26,851 | 26,288 | 29,655 | 29,320 | 26,909 | 26,342 |
|  | Europe and Turkey | 0 | 0 | 0 | 0 | 0 | 0 | 0 | 0 |
|  | Africa | 3,914 | 3,950 | 3,521 | 3,017 | 3,960 | 3,981 | 3,664 | 3,142 |
|  | Northern, Central, Western Asia | 0 | 0 | 0 | 0 | 0 | 0 | 0 | 0 |
|  | South and South-East Asia | 2,113 | 2,069 | 1,864 | 1,790 | 2,008 | 1,956 | 1,749 | 1,674 |
|  | Eastern Asia and Oceania | 94 | 85 | 59 | 71 | 89 | 80 | 51 | 66 |
| Birds | Global | 1,106,039 | 1,097,183 | 974,642 | 945,435 | 1,078,496 | 1,066,065 | 944,398 | 916,237 |
|  | North America | 93,200 | 89,403 | 83,922 | 85,009 | 90,079 | 85,448 | 79,208 | 80,412 |
|  | South and Central America | 744,546 | 731,691 | 636,381 | 621,926 | 736,742 | 721,791 | 626,967 | 612,773 |
|  | Europe and Turkey | 6,505 | 6,729 | 5,919 | 6,109 | 5,513 | 5,531 | 4,648 | 4,799 |
|  | Africa | 66,882 | 67,614 | 61,078 | 51,699 | 64,820 | 65,529 | 60,337 | 51,425 |
|  | Northern, Central, Western Asia | 58,244 | 67,543 | 61,744 | 61,611 | 56,836 | 64,726 | 58,931 | 58,677 |
|  | South and South-East Asia | 108,111 | 103,081 | 93,208 | 90,151 | 98,611 | 94,558 | 84,890 | 81,830 |
|  | Eastern Asia and Oceania | 28,552 | 31,121 | 32,391 | 28,931 | 25,897 | 28,481 | 29,418 | 26,319 |
| Mammals | Global | 463,049 | 463,939 | 405,196 | 383,905 | 461,800 | 463,170 | 406,990 | 386,476 |
|  | North America | 19,958 | 19,203 | 17,441 | 17,931 | 20,352 | 19,437 | 17,617 | 18,130 |
|  | South and Central America | 295,131 | 290,609 | 248,470 | 241,019 | 292,463 | 287,551 | 245,894 | 238,440 |
|  | Europe and Turkey | 817 | 884 | 689 | 711 | 921 | 966 | 765 | 782 |
|  | Africa | 83,610 | 85,414 | 76,395 | 64,693 | 81,037 | 82,872 | 75,639 | 64,490 |
|  | Northern, Central, Western Asia | 19,231 | 22,281 | 19,964 | 19,995 | 21,890 | 26,034 | 24,233 | 24,382 |
|  | South and South-East Asia | 31,997 | 32,469 | 28,906 | 27,526 | 32,538 | 32,965 | 29,306 | 27,976 |
|  | Eastern Asia and Oceania | 12,305 | 13,078 | 13,331 | 12,030 | 12,600 | 13,343 | 13,536 | 12,275 |
| Taxa combined | Global | 1,605,574 | 1,597,210 | 1,412,759 | 1,361,152 | 1,576,764 | 1,565,236 | 1,384,302 | 1,334,496 |
|  | North America | 113,980 | 109,343 | 101,987 | 103,586 | 111,186 | 105,549 | 97,367 | 99,101 |
|  | South and Central America | 1,069,220 | 1,051,548 | 911,702 | 889,233 | 1,058,859 | 1,038,662 | 899,769 | 877,555 |
|  | Europe and Turkey | 7,321 | 7,613 | 6,608 | 6,820 | 6,434 | 6,497 | 5,412 | 5,582 |
|  | Africa | 154,406 | 156,977 | 140,994 | 119,409 | 149,817 | 152,383 | 139,640 | 119,058 |
|  | Northern, Central, Western Asia | 77,475 | 89,824 | 81,708 | 81,606 | 78,726 | 90,761 | 83,164 | 83,059 |
|  | South and South-East Asia | 142,221 | 137,619 | 123,977 | 119,466 | 133,156 | 129,480 | 115,945 | 111,480 |
|  | Eastern Asia and Oceania | 40,951 | 44,285 | 45,781 | 41,033 | 38,586 | 41,905 | 43,005 | 38,661 |
